# Identification and evaluation of a pinocembrin analog as a TRPV1 inhibitor with analgesic properties in murine pain models

**DOI:** 10.3389/fphar.2025.1585181

**Published:** 2025-06-10

**Authors:** Hanbin Chen, Guanghong Li, Lin Deng, Nan Xu, Simon Ming-Yuen Lee, Xiaowei Nie, Jin-Song Bian

**Affiliations:** ^1^ Department of Pharmacology, Joint Laboratory of Guangdong-Hong Kong Universities for Vascular Homeostasis and Diseases, School of Medicine, Southern University of Science and Technology, Shenzhen, China; ^2^ SUSTech Homeostatic Medicine Institute, School of Medicine, Southern University of Science and Technology, Shenzhen, China; ^3^ Department of Cardiology, The Eighth Affiliated Hospital of Sun Yat-sen University, Shenzhen, Guangdong, China; ^4^ State Key Laboratory of Quality Research in Chinese Medicine and Institute of Chinese Medical Sciences, University of Macau, Macao, China; ^5^ Department of Food Science and Nutrition, The Hong Kong Polytechnic University, Hung Hom, Hong Kong, China; ^6^ PolyU-BGI Joint Research Centre for Genomics and Synthetic Biology in Global Ocean Resources, The Hong Kong Polytechnic University, Hung Hom, Hong Kong, China; ^7^ Research Centre for Chinese Medicine Innovation, The Hong Kong Polytechnic Universityy, Hung Hom, Hong Kong, China; ^8^ State Key Laboratory of Chemical Biology and Drug Discovery, The Hong Kong Polytechnic University, Hung Hom, Hong Kong, China; ^9^ Research Institute for Future Food, The Hong Kong Polytechnic University, Hung Hom, Hong Kong, China; ^10^ Research Institute for Smart Ageing, The Hong Kong Polytechnic University, Hung Hom, Hong Kong, China; ^11^ Department of Human Cell Biology and Genetics, School of Medicine, Southern University of Science and Technology, Shenzhen, China

**Keywords:** virtual screening, molecular dynamics simulation, PINO analogue, analgesic, antiinflammation

## Abstract

**Introduction:**

Pain is a complex phenomenon involving physiological and psychological responses to noxious stimuli. Long-term opioid or NSAID use leads to reduced efficacy and tolerance. Initially a thermosensitive receptor, TRPV1 is increasingly recognized as a target for analgesic intervention.

**Methods:**

Our investigation is focused on the exploration of novel TRPV1 antagonists derived from natural sources through computational screening methodologies, aiming to assess their efficacy as analgesic agents.

**Results:**

Among the compounds screened, a promising TRPV1 antagonist named pinocembrin-7-o-3-o-galloyl-4-6-hexahydroxydiphenoyl-beta-d-glucoside (PINO) has exhibited superior stability in its interaction with TRPV1 through virtual screening and molecular dynamics simulation. A dosage of 20 mg/kg of PINO had been shown to reduce the writhing response in acetic acid-induced mice, elevate the thermal pain threshold in the hot water tail-flick and hot plate assays, and concurrently increase the mechanical pain threshold in CFA-induced inflammatory pain models in mice. Moreover, in a murine Lewis lung carcinoma cell line LL-induced bone cancer pain model, PINO also effectively raised the thermal and mechanical pain thresholds in mice. Furthermore, PINO had been found to attenuate the production and gene expression of pro-inflammatory cytokines. The underlying mechanism was attributed to the suppression of NF-κB and MAPK signaling cascades.

**Discussion:**

This innovative compound represents a prospective avenue for the management of acute, chronic, and bone cancer pain, providing a viable alternative analgesic option for individuals suffering from such conditions.

## 1 Introduction

Pain represents an aversive sensory and affective phenomenon associated with real or potential harm to bodily tissues (De Ridder et al., 2021). The market size for pain management is forecasted to rise from 78.12 billion in 2024 to 93.2 billion in 2029, demonstrating a compound annual growth rate of 3.59% throughout the projected period (2024–2029) (Advisory, 2024). Traditional pain management approaches frequently encompass the utilization of analgesics, such as opioids, nonsteroidal anti-inflammatory drugs (NSAIDs), and other pharmaceuticals. Nevertheless, these treatments often present limitations, including reduced efficacy, notable side effects, and the risk of addiction, especially in the case of opioids (Alorfi, 2023).

The transient receptor potential vanilloid 1 (TRPV1) channel is classified under the vanilloid subfamily of TRP channels, initially identified as a heat-sensing receptor in the pain pathway (Caterina et al., 1997). TRPV1 plays a pivotal role in pain perception as a non-selective cation channel and is activated by diverse stimuli like capsaicin, heat, and pro-inflammatory agents (Xia et al., 2011). This inhibition of TRPV1 takes place via interaction with the vanilloid-binding pocket, thereby stabilizing the channel in a closed conformation to prevent activation by noxious stimuli (Arnold et al., 2024). Consequently, modulating TRPV1 activity with antagonists has the potential to alleviate pain without the side effects commonly associated with traditional opioid analgesics, known for their addictive properties and other adverse effects (Goldmann et al., 2015). Hence, antagonists targeting TRPV1 have become prominent focal points in the advancement of innovative analgesics, especially concerning chronic pain management (Andrei et al., 2022).

Natural products have proven to be a significant source of bioactive compounds. Owing to their structural heterogeneity, natural products play an important role in the contemporary transition from empirical drug screening to systematic drug design (Nisbet and Moore, 1997). Integrating virtual screening with natural product libraries can greatly accelerate the discovery of new TRPV1 antagonists (Sadybekov and Katritch, 2023). This method identifies new candidates for pain management and enables exploration of the pharmacological profiles of natural products, which are frequently well-characterized for their safety and efficacy owing to their historical use in traditional medicine (Atanasov et al., 2021).

This study aims to discover new TRPV1 antagonists from natural products through virtual screening and evaluate their analgesic effects. We identified a new TRPV1 antagonist, pinocembrin-7-o-3-o-galloyl-4-6-hexahydroxydiphenoyl-beta-d-glucoside (PINO), which exhibited a more stable interaction with TRPV1 compared to other candidate compounds, and its inhibitory effect was confirmed through calcium imaging. PINO demonstrated a remarkable analgesic effect in various types of pain models, and its potential mechanism may be associated with its anti-inflammatory properties. In conclusion, virtual screening of TRPV1 antagonists represents a state-of-the-art strategy in drug discovery that integrates computational techniques with experimental validation to identify novel therapeutic agents for pain management. The discovery of PINO has the potential to effectively modulate TRPV1 activity, providing new hope for patients with pain and related disorders.

## 2 Materials and methods

### 2.1 Ligand preparation

The natural product library containing 127,719 small molecules was downloaded from ZINC (Irwin and Shoichet, 2005), a comprehensive repository of commercial compounds for virtual screening. The compound structure format (mol2 or sdf) was converted into the pdb format using Gypsum-DL 1.2.1 (Ropp et al., 2019a; Ropp et al., 2019b). Gypsum-DL is complimentary and open-source software specifically designed for preparing 3D small molecules. Subsequently, the compound structures in pdb format were converted to pdbqt format using the prepare_ligand4. py packages from AutoDockTools (Sanner, 1999).

### 2.2 Protein preparation and free energy landscape

The 3D structure of TRPV1 (PDB ID: 5IS0) was downloaded from Protein Data Bank (http://www.rcsb.org/pdb) (Neuberger et al., 2023). The water and the small molecules were removed from the structure. To obtain the most stable structure of TRPV1 under physiological conditions, molecular dynamics (MD) simulation was employed to predict the optimal conformation. GROMACS is a software tool primarily used for conducting dynamic simulations of biomolecules (Abraham et al., 2015). In this study, GROMACS was employed to generate the free energy landscape (FEL) and calculate the Gibbs free energy to predict the most stable structure. Briefly, The structure of TRPV1 was submitted to CHARMM-GUI for protein preparation (Jo et al., 2008). The transmembrane was incorporated using POPC (Wu et al., 2014). Water and 150 mL NaCl were introduced to neutralize the ion balance. Subsequently, the system underwent energy minimization for 5 × 10^5^ steps. Following the completion of 1.2 × 10^6^ steps of the canonical ensemble (NVT) and isothermal-isobaric (NPT) equilibration, a 200 ns MD simulation was conducted using a charmm36 m forcefield (Huang et al., 2017). The root mean square deviation (RMSD) and radius of gyration (Rg) were extracted from the MD simulation trajectory. Subsequently, the gmx sham command line was used to analyze the relationship between RMSD and Rg, resulting in the generation of the FEL. The FEL provides insights into the stability of the various conformations assumed by a protein (Gupta et al., 2019). The Gibbs free energy, which is a function dependent on enthalpy and entropy, serves as a benchmark for the stability of a protein (Aier et al., 2016). The optimal conformation was determined using the FEL.

### 2.3 High throughput virtual screening

**FIGURE 1:**
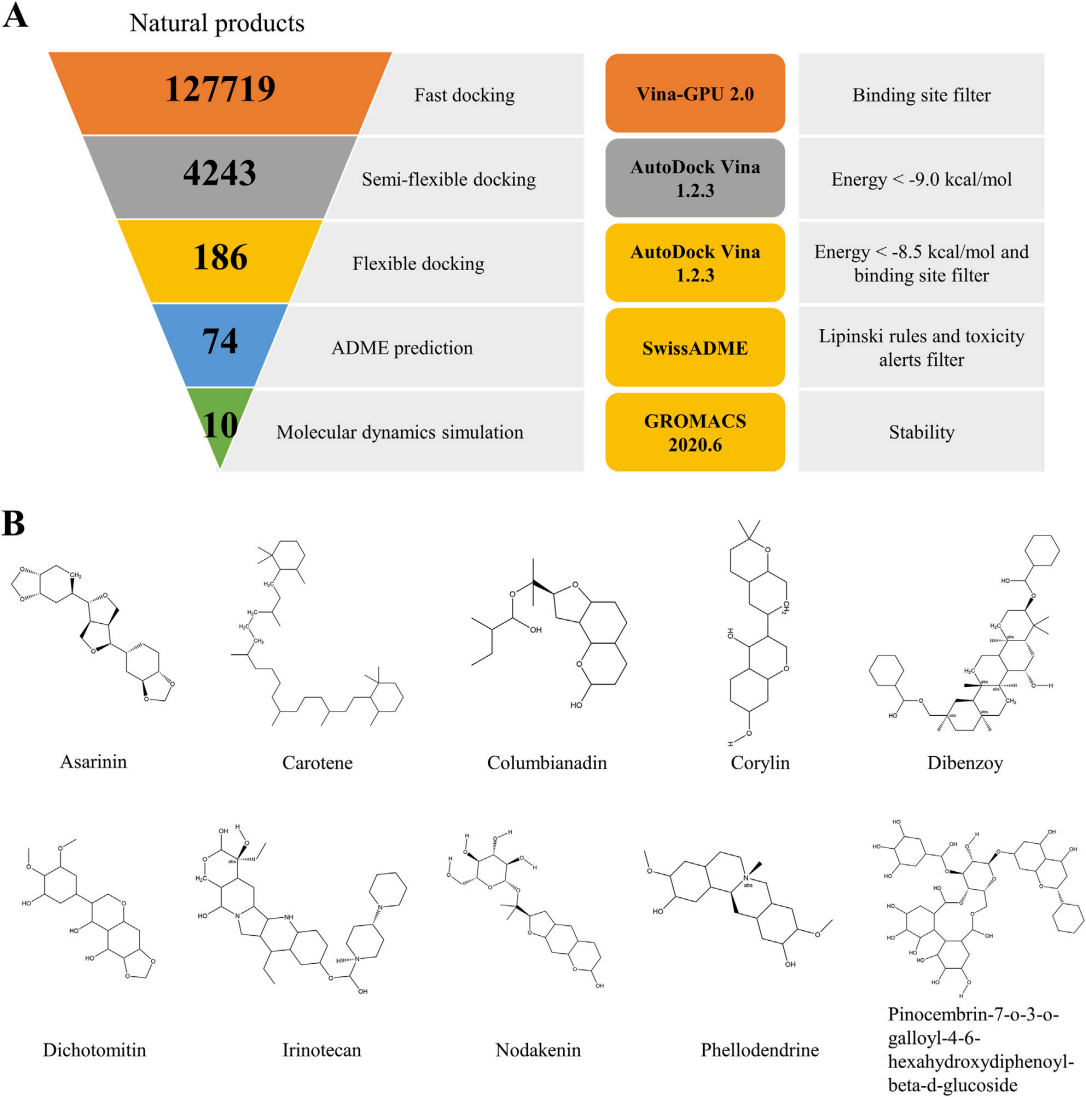
The virtual screening workflow **(A)**. Chemical structures of the nine compounds identified by virtual screening **(B)**.

The custom high-throughput virtual screening pipeline is depicted in Figure 1. In brief, a total of 127719 small molecules and TRPV1 were subjected to docking using Vina-GPU and AutoDock Vina 1.2.5 (Eberhardt et al., 2021; Trott and Olson, 2010; Ding et al., 2023). The active pocket was defined based on the position of the TRPV1 inhibitor capsazepine from the protein structure. The TRPV1 grid box encompassed dimensions of 30 × 30 × 30 grid points, with the central coordinates set at x = 109.944, y = 93.765, and z = 104.958. The specified parameters included exhaustiveness = 32, energy_range = 4, and num_modes = 10. The top 10% scores of the compounds were selected, and molecules whose binding sites were not located in the active pocket were excluded. Subsequently, the small molecules underwent triple docking to reduce software errors. Following this, the amino acids within a 5-angstrom radius of the active pocket were designated as flexible amino acids, and specific compounds were selected for flexible docking. Subsequently, the compounds underwent ADME prediction using the SwissADME server and were filtered according to the Lipinski rules and toxicity alerts (Daina et al., 2017). Ultimately, ten compounds passed the ADME prediction and were selected based on the highest docking scores.

### 2.4 Molecular dynamics simulation

MD simulation was utilized to predict the binding characteristics between TRPV1 and the compounds. Ten complexes of compounds with TRPV1 were subjected to the High-Throughput Simulator available on CHARMM-GUI (Guterres et al., 2022). The generated topology file of the ligand was prepared using CGenFF (Vanommeslaeghe et al., 2010). In addition, the known TRPV1 antagonists capsazepine (CPZ) were docking with TRPV1 using AutoDock Vina 1.2.3 with the same condition mentioned in section 2.3. The TRPV1-CPZ complexes were subjected to MD simulation to compare the selected compounds’ similarities and differences. This procedure followed a similar protocol as described in section 2.2. Following the MD simulation, the RMSD values of the ligands and protein, the number of hydrogen bonds, and the solvent-accessible surface area (SASA) between TRPV1 and the compounds were calculated and analyzed. The principal component analysis (PCA) combined with free energy landscape (FEL) was employed to determine the conformational dynamics during MD simulation. The binding free energy was evaluated through the MMPBSA method employing the gmx_MMPBSA tool (https://github.com/Jerkwin/gmxtool/tree/master/gmx_mmpbsa, accessed on 4 April 2022) (Valdés-Tresanco et al., 2021; Li, 2022).

### 2.5 Materials

All selected candidate TRPV1 antagonist compounds were obtained from Topscience, Shanghai, China (www.tsbiochem.com) without further purification. Complete Freund’s adjuvant (CFA) and Fluo-4 were obtained from Sigma-Aldrich (Saint Louis, United States). Acetic acid was obtained from Shanghai Aladdin Biochemical Technology (Shanghai, China). Capsaicin, chloroform, isopropanol, and ethanol were acquired from Shanghai Macklin Biochemical Technology (Shanghai, China). Capsazepine and normal saline were purchased from Techisun Bio-technology (Shenzhen, China). Penicillin/streptomycin was acquired from Proteintech Group, Inc. (Wuhan, China), while fetal bovine serum (FBS) was sourced from ExCell Bio (Shanghai, China). Dulbecco’s modified Eagle medium with high glucose (4.5 g/L) was procured from Thermo Fisher Scientific (Shanghai, China). RPMI1640 medium was procured from Gibco-BRL (Gaithersburg, United States). RNA extraction reagent RNAiso Plus was purchased from Takara Biomedical Technology (Dalian, China). cDNA Synthesis Kit and qPCR SYBR Green were purchased from Yeasen Biotechnology (Shanghai, China). Mouse interleukin-1 beta (IL-1β), interleukin-6 (IL-6), and tumor necrosis factor-alpha (TNF-α) Valukine ELISA were purchased from Novus Biologicals (Littleton, United States). RIPA lysis buffer with a protease inhibitor phenylmethanesulfonyl fluoride (PMSF), protease and phosphatase inhibitor cocktail for general use, BCA protein assay kit, QuickBlock™ Protein-Free Blocking Buffer for Western blot and stripping buffer were obtained from Beyotime Biotechnology (Nantong, China). Polyvinylidene difluoride (PVDF) membrane and Immobilon Western HRP substrate were purchased from Millipore (Burlington, United States). Phospho-NF-κB p65 (Ser468) antibody, NF-κB p65 antibody, Phospho-IκB Alpha (Ser32/36) antibody, IκB Alpha antibody, Phospho-p38 MAPK (Thr180/Tyr182) antibody, p38 MAPK antibody, Phospho-JNK (Tyr185) antibody, JNK antibody, Phospho-ERK1/2 (Thr202/Tyr204) antibody, ERK1/2 antibody, TRPV1 antibody, β-actin antibody, glyceraldehyde 3-phosphate dehydrogenase (GAPDH) antibody, β-Tubulin and Goat Anti-Rabbit IgG (H + L) were purchased from Proteintech Group, Inc. (Wuhan, China).

### 2.6 Calcium imaging

10% fetal bovine serum and 1% penicillin-streptomycin in DMEM medium were used to culture the human embryonic kidney (HEK293) cells. To establish human TRPV1-expressing HEK293 cells, the Lipo8000TM Transfection Reagent was employed. For transfection, 2.5 μg of plasmid DNA containing the human TRPV1 protein (NM_018727.5, IGE Biotechnology, Guangzhou, China) was introduced into the HEK293 cells. After seeding the cells on a 12-well plate, they were incubated at 37°C and 5% CO_2_ for 24 h. To evaluate compound effects, cells were pre-treated with different concentrations of test compounds or CPZ (10 μM) for 1 h. Unbound compounds were then removed by triple washing with HEPES buffer, leaving only channel-bound antagonists. Subsequently, the cells were stained with 2 μM Fluo-4/AM, dissolved in HEPES buffer, and cocultured in a dark environment for 30 min. After three additional HEPES washes to remove residual dye, cells were stimulated with capsaicin (applied alone in HEPES buffer) to induce calcium influx. Calcium fluorescence was promptly measured utilizing the cellSens imaging system integrated into an IX73 microscope manufactured by Olympus Co. in Tokyo, Japan. The excitation and emission wavelengths utilized for this measurement were 494 nm and 516 nm, respectively. The fluorescence changes were analyzed using CellSens software. Sixteen cells were randomly selected, and the actual fluorescence intensity was determined by subtracting background signals from cellular fluorescence measurements. The blank control group served as the baseline, and fluorescence intensities from different experimental groups were normalized to this baseline by calculating fold-change values (experimental group fluorescence/blank control fluorescence).

### 2.7 Animals

Male C57BL/6J mice were procured from GemPharmatech (Nanjing, China) for the study. The use of male mice was consistent across all experiments conducted. The mice were allocated randomly to the experimental cohorts. Throughout the research period, the mice were accommodated in a regulated environment following a 12-h light-dark cycle, with illumination commencing at 7 a.m. The ambient temperature was maintained between 23°C and 25°C, ensuring the comfort of the animals. They had unrestricted access to both water and standard laboratory chow. The mice were kept in a specific pathogen-free (SPF) facility to uphold their health and welfare. All animal experiments were conducted strictly with the protocols approved by the Institutional Animal Care and Use Committee of the Southern University of Science and Technology. Stringent adherence to the relevant protocols and guidelines governing the ethical treatment of animals was maintained throughout the study.

### 2.8 Animal behavior

The doses of CPZ (20 mg/kg) and PINO (20 mg/kg) were selected based on systematic dose optimization experiments. A pilot dose-response study was conducted in the CFA-induced inflammatory pain model using PINO at 10, 15, and 20 mg/kg (intraperitoneal administration; n = 16–18/group). The 20 mg/kg dose demonstrated maximal and sustained analgesic efficacy compared to vehicle controls. CPZ was administered at an equivalent dosage (20 mg/kg) to enable direct therapeutic comparison while controlling for potential concentration-dependent confounding effects between the two compounds.

#### 2.8.1 Body temperature

To evaluate the thermoregulatory effects of the TRPV1 inhibitor, 6 mice were individually identified. After acclimatization, baseline rectal temperature was measured using a digital thermometer. PINO (20 mg/kg, 0.1 mL/10 g body weight) was administered via intraperitoneal injection. Post-administration temperatures were recorded at 0, 1, 2, 4, 6, 8, 12 and 24 h. The temperature differentials (ΔT) were calculated as the difference from baseline values.

#### 2.8.2 Acetic acid-induced stretching

Firstly, 32 mice were divided into four groups: the control group was pretreated with saline without intraperitoneal injection (i.p.) with 0.6% acetic acid; the vehicle group was pretreated with saline and i. p. with 0.6% acetic acid; Other two groups were pretreated with capsazepine (20 mg/kg) or PINO (20 mg/kg) and i. p. with 0.6% acetic acid. The drugs were i. p. 1 h before being treated with 0.6% acetic acid. Every mouse was placed in a PMMA enclosure and given 20 min to acclimate to the surroundings. Subsequently, observations would commence 5 min post acetic acid injection. A stretch was operationally characterized as an abdominal contraction succeeded by hind limb extension (Bagdas et al., 2016). The stretch times were recorded for 20 min.

#### 2.8.3 Hot water tail-flick test

The analgesic effect of PINO (20 mg/kg; i. p.) was assessed using the hot water tail-flick test. Mice were delicately swathed in a soft cloth and their tail tips were submerged in a 50°C water bath to record the latency of tail flicking. Each assessment consisted of three trials with an intertrial interval of 3–4 min. During the intervals, the mice were returned to their designated home enclosures (Zhang et al., 2018).

#### 2.8.4 Hot plate test

The impact of PINO (20 mg/kg; i. p.) and CPZ (20 mg/kg; i. p.) was assessed using a hot plate thermal nociceptive test, applying temperature settings and experimental conditions established in prior investigations (Neelakantan et al., 2015). The latency, defined as the duration until the mouse licked its hind paw, leaped off the hot plate, or reached a cut-off time of 25 s, was recorded. Two baseline latency measurements were taken with a 5-min interval between each, and the average value was considered as the baseline latency measure for each mouse. Subsequently, the mice were administered PINO and CPZ at 20 mg/kg each, followed by the assessment on the hot plate after 1 h.

#### 2.8.5 CFA-induced inflammatory pain

CFA-induced inflammatory pain represents a well-established model of persistent inflammatory hyperalgesia established by Larson et al. (1986). The standard protocol involves the injection of 20 μL of CFA into the plantar surface of the right hind paw to elicit inflammation. The mice were randomly allocated into seven distinct groups: Group 1 served as the control group with normal saline, Group 2 as the CFA-induced inflammation group, Group 3 as the CPZ group treated with CFA, and Group 4–6 as the PINO (at doses of 10, 15, and 20 mg/kg) treatment groups alongside CFA administration. Group 7 received PINO (20 mg/kg) without CFA induction. Mechanical allodynia, as evaluated through the von Frey test, was assessed both before and 7 days post-CFA injection. The mechanical response threshold of escape behavior in mice was evaluated using a series of ascending von Frey filaments (North Coast Medical Inc., CA, United States), commencing with the smallest filament having a bending force of 0.16 g. Before testing, the mice were habituated in individual 6 × 6 cm boxes on an elevated wire grid for 30 min daily over three consecutive days. On the day of the experimental procedure, after a 30-min acclimation period to the testing environment, the hind paws of the mice were stimulated with von Frey filaments of logarithmically increasing stiffness (ranging from 0.16 to 2.56 g) applied perpendicularly to the central plantar surface. A positive response was recorded when the mouse reacted to the filament, with the corresponding force being documented; conversely, a lack of response signified a negative result, prompting the application of the next larger filament. The mechanical withdrawal threshold was quantified in grams (g) utilizing the prescribed formula (Chaplan et al., 1994), denoting the pressure exerted by the von Frey filament that elicited a response from the animal.

#### 2.8.6 Bone cancer pain model

The bone cancer pain model was developed by the methodology described in a prior publication (Wang K. et al., 2020). The murine Lewis lung carcinoma cell line LLC1 (ATCC^®^ CRL-1642) was obtained from the American Type Culture Collection (ATCC). The cells were cultured in Dulbecco’s modified Eagle medium with high glucose comprising 10% fetal bovine serum and 1% penicillin/streptomycin solution at 37°C in a 5% CO_2_ atmosphere.

To establish the bone cancer pain model, the LLC1 cell line was dissociated using 0.05% trypsin and subsequently suspended at a concentration of 5 × 10^7^ cells/mL in phosphate-buffered saline (PBS). Mice were anesthetized with 2.5% isoflurane, and a superficial incision measuring 0.5–1 cm was made near the knee to expose the patellar ligament. A 25-gauge needle was inserted at the intercondylar notch of the left femur into the femoral cavity. This needle was then replaced with a 10 μL microinjection syringe containing a 4 μL suspension of tumor cells (2 × 10^5^) and 2 μL of an absorbable gelatin sponge solution to seal the injection site. The contents of the syringe were injected slowly into the femoral cavity over 2 min. To prevent the leakage of tumor cells from the bone cavity, the external injection site was sealed with silicone adhesive (Kwik-Sil, World Precision Instruments). Animals that experienced unsuccessful injections or exhibited impaired mobility post-surgery were excluded from the study.

Subsequently, to evaluate the protective effects against bone cancer pain, treatment with PINO or CPZ (20 mg/kg, intraperitoneally) was administered on day 7 post-inoculation. Bone pain was assessed through thermal and mechanical pain tests, specifically utilizing the hot plate and von Frey tests.

### 2.9 Assessment of inflammatory cytokines by ELISA

Following 7 days post-CFA injection, behavioral tests were conducted to confirm the successful establishment of the models. Subsequently, the mice were euthanized via cervical dislocation and then decapitated. Blood was collected through cardiac puncture, allowed to coagulate at room temperature for 30 min, and then centrifuged at 2000 × g for 10 min at 4°C to obtain the supernatant (serum). ELISA procedures were performed according to the manufacturer’s guidelines, and a standard curve was generated for each experiment.

### 2.10 Assessment of mRNA expression of DRG

7 days after the injection of CFA, behavior tests were performed to verify the successful construction of the models. Subsequently, the mice were euthanized by cervical dislocation, followed by decapitation. The dissections were performed on ice. The dissected L4–L6 dorsal root ganglia (DRG) were then isolated, and the connective tissues were meticulously removed. Following the dissection, the DRG tissues were promptly snap-frozen and preserved at −80°C. Total RNA extraction was carried out using RNAiso Plus. Approximately 1 μg of RNA was reverse transcribed into cDNA using a reverse transcription kit, following the manufacturer’s protocol. Subsequently, qPCR was conducted with the SYBR Green PCR kit. All primer sequences designed for the study are provided in the Supplementary Material. PCR was carried out with an initial denaturation step at 95°C for 30 s, followed by 40 amplification cycles consisting of 60°C for 30 s, 72°C for 30 s, 95°C for 10 s, and 65°C for 5 s each. Normalization of all data was performed relative to the mRNA expression of 18S rRNA, serving as the internal control. The results for the target gene were quantified and presented utilizing the 2^−ΔΔCT^ method (Livak and Schmittgen, 2001).

### 2.11 Cell culture

The RAW264.7 macrophage cell line (ATCC TIB-71) was acquired from ATCC. The RAW264.7 cells were maintained in RPMI1640 medium supplemented with 10% (v/v) FBS and 1% (v/v) penicillin-streptomycin. To examine the toxicity of PINO, the RAW264.7 cells were seeded into 96-well plate. Different concentrations (ranging from 6.25 to 100 μM) of PINO were added to wells. The toxicity of the PINO was determined by the MTT assay after a 24-h incubation.

### 2.12 Assessment of mRNA expression of RAW264.7

The RAW264.7 cells were seeded in a 6-well plate and incubated for 24 h to allow adherence and growth. Subsequently, the cells were treated with varying concentrations ranging from 2.5 to 10 μM of PINO and co-cultured for 1 h. Subsequently, TNF-α was added to achieve a final concentration of 10 ng/mL, and the cells were incubated for an additional 1 h. Cell mRNA was extracted using RNAiso plus according to the procedures outlined in section 2.11 of the protocol. The primer sequences were provided in the Supplementary Material.

### 2.13 Western blotting

RAW264.7 cells were co-incubated with 10 μM of PINO for 1 h. Subsequently, TNF-α was then added to reach a final concentration of 10 ng/mL, followed by co-culturing for an additional 1 h. The cells were washed three times with PBS and then lysed using RIPA buffer (containing 1% PMSF and 1% phosphatase inhibitor cocktail) for 20 min on ice. The lysate was centrifuged at 13,000xg for 15 min at 4°C. Protein concentrations in the supernatant were assessed using the BCA assay to ensure standardization across all experimental groups. The samples were electrophoresed on a 10% sodium dodecyl sulfate-polyacrylamide gel (SDS-PAGE) and subsequently transferred to PVDF membranes, followed by blocking with QuickBlock blocking buffer. Immunoblot analysis was conducted by incubating the samples with antibodies for p-IκBα, IκBα, p-p65, p65, p-p38, p38, p-ERK1/2, ERK1/2, p-JNK, and JNK. GAPDH, TRPV1, β-actin, GADPH, and β-Tubulin were utilized as internal loading controls. To investigate TRPV1 dependency, cells were co-treated with PINO (10 μM) and capsaicin (10 μM) for 1 h. Immunoblot analysis was conducted by incubating the samples with antibodies for p-IκBα, IκBα, p-p65, p65, p-p38, p38, p-ERK1/2, ERK1/2. GADPH were utilized as internal loading controls.

### 2.14 Quantification and statistical analysis

The analysis of data and statistics was performed using GraphPad Prism version 8.3. The results are expressed as mean ± standard deviation (SD). And the figures indicated significance values. Comprehensive statistical details for the experiments are available in the figure captions of each respective figure. Detailed statistical information for the experiments can be found in the figure captions of each corresponding figure. To compare the means between the two groups, the unpaired Student’s t-test was employed. In cases involving more than two groups, a one-way ANOVA with Bonferroni *post hoc* adjustment for multiple comparisons was utilized.

## 3 Results

### 3.1 Virtual screening and initial hit evaluation

The ultimate aim of the present study was to identify novel and specific TRPV1 inhibitors and evaluate their analgesic effect. The virtual screening workflow is presented in Figure 1A. Initially, the most stable structure of TRPV1 was obtained through the free energy landscape (FEL) (Supplementary Figure S1). The active site chosen for docking was identified as the primary drug-binding pocket. The ZINC natural product library of 127718 phytochemicals was subjected to Vina-GPU 2.0 to perform docking against TRPV1. Filtered by the binding site, this led to a subset of 4,243 at the end of the docking. Subsequently, a semi-flexible docking approach using AutoDock Vina 1.2.3 was applied to the 4,243 candidate compounds. From these, 4,243 potential hits were identified based on a scoring criterion below the specified threshold of −9.0 kcal/mol. Next, the remaining compounds were subjected to flexible docking with AutoDock Vina 1.2.3. After filtration based on the binding site and a score below the defined threshold of −8.5 kcal/mol, a subset of 186 phytochemicals emerged from the flexible docking phase (Supplementary Figure S2). These compounds then underwent ADME (Absorption, Distribution, Metabolism, Excretion) prediction. Following filtration based on Lipinski’s rules and toxicity alerts, the top 10 compounds with the highest docking scores were selected for molecular dynamics simulations using GROMACS 2020.6 to investigate the stability of binding interactions between TRPV1 and the compounds. The chemical structures of the chosen compounds are depicted in Figure 1B and Table 1.

**TABLE 1:** List of the top 10 screened compounds.

Name	CAS	Selected plant	Category
Asarinin	133‐04‐0	*Asarum sieboldii Miq*	Lignans
Beta-Carotene	7,235‐40‐7	*Raphanus sativus*	Terpenoids
Columbianadin	5,058‐13‐9	*Eleutherococcus senticosus*	Terpenoids
Corylin	53,947‐92‐5	*Psoraela corylifolia*	Flavonoids
3,29-Dibenzoyl Rarounitriol	873,001‐54‐8	*Trichosanthes kirilowii Maxim*	Terpenoids
Dichotomitin	88,509‐91‐5	*Belamcanda chinensis*	Others
Irinotecan	100,286‐90‐6	*Camptotheca acuminata*	Alkaloids
Nodakenin	495‐31‐8	*Angelica biserrata*	Coumarins
Phellodendrine	6,873‐13‐8	*Phellodendron chinense*	Alkaloids
Pinocembrin-7-o-3-o-galloyl-4-6-hexahydroxydiphenoyl-beta-d-glucoside (PINO)	205,370‐59‐8	The herbs of *Lindera strychnifolia*	Flavonoids

### 3.2 Molecular dynamics (MD) analysis of the protein-ligand complexes of the top 10 phytochemical inhibitors

Molecular dynamics (MD) is a ‐computational methodology employed to examine the dynamic complexities of intricate systems, wherein atoms and molecules interact and evolve temporally (Oyedele et al., 2022). In order to evaluate the stability of the docked complexes, a 100 ns MD simulation was conducted on the protein-ligand complexes involving the top ten inhibitors with TRPV1.

#### 3.2.1 Root mean square deviation (RMSD) of ligand

**FIGURE 2:**
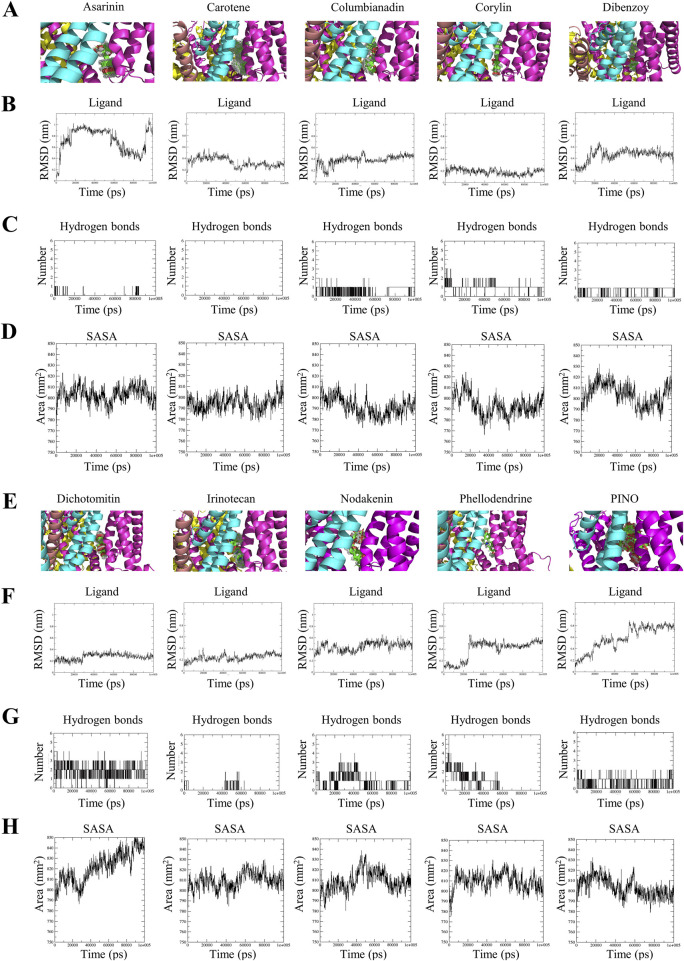
The superimposed trajectories of TRPV1 with asarinin, carotene, columbianadin, corylin, dibenzoy, dichotomitin, irinotecan, nodakenin, phellodendrine and PINO during the simulation period **(A,E)**, the RMSD values of the small molecules **(B,F)**, the number of hydrogen bonds formed between the small molecules and TRPV1 **(C,G)**, and the SASA of TRPV1 **(D,H)**.

The superimposition of ligand-protein complexes at various simulation time points is illustrated in Figures 2A,E. Hence, comparing the conformation of protein-ligand complexes at different time points up to 100 ns offers valuable structural insights that facilitate the comprehension of potential changes in the ligand’s pose (Bibi et al., 2022). The RMSD of the backbone atoms concerning the ligand was utilized to evaluate the structural stability of the ligand-protein complexes. The RMSD serves as a metric to indicate the extent of conformational changes in a structure over time (Oyedele et al., 2022). The analysis in Figures 2B,F reveals that the most stable compounds among the hits are corylin, dichotomitin, and irinotecan. Upon combining superimposition and RMSD analysis of the ligand, these three compounds exhibited minor deviations in structural conformation.

The PINO comprises three chemical constituents: pinocembrin, gallic acid, and hexahydroxydiphenoyl (HHDP). The superimposition revealed the stability of HHDP, whereas both pinocembrin and gallic acid displayed significant fluctuations throughout the simulation period. Detailed analysis of PINO’s MD trajectories revealed distinct behaviors among its three constituent groups (Supplementary Video S1). The HHDP moiety remained stably embedded within the TRPV1 binding pocket, exhibiting minimal RMSD fluctuations, while the pinocembrin and glucoside groups displayed significant mobility. This suggests that HHDP serves as the primary anchor for binding, whereas the flexibility of peripheral groups may facilitate initial ligand recognition.

#### 3.2.2 Hydrogen bond number of ligand-protein complexes

Hydrogen bonds and their varying strengths within an aqueous milieu play a pivotal role in protein-ligand interactions, particularly in scenarios where the mechanism of action revolves around hydrolysis, where water assumes a central role in the chemical degradation process (Rui et al., 2022). The average number of intermolecular hydrogen bonds formed between asarinin-TRPV1, carotene-TRPV1, columbinadin-TRPV1, corylin-TRPV1, dibenzoy-TRPV1, dichotomintin-TRPV1, irinotecan-TRPV1, nodakenin-TRPV1, phellodendrine-TRPV1, and PINO-TRPV1 were as follows: 0.048, 0, 0.530, 1.068, 0.874, 1.951, 0.146, 1, 0.836, and 0.800, respectively. This evidence is also supported by the graphical representation of H-bonds in Figures 2C,G. This result indicates that corylin, dibenzoy, nodakenin, phellodendrine, and PINO formed a higher number of hydrogen bonds with TRPV1.

#### 3.2.3 Solvent accessible surface area (SASA)

SASA is a valuable analysis technique for determining changes in protein accessibility to solvent (Bibi et al., 2022). Greater values of SASA indicate a more expanded or open structure that has been subjected to increased solvent exposure, while lower values suggest the opposite (Mazola et al., 2015). The results indicated that the binary complexes of asarinin-TRPV1 (803.246 nm^2^), carotene-TRPV1 (794.923 nm^2^), columbinadin-TRPV1 (791.872 nm^2^), corylin-TRPV1 (793.783 nm^2^), dibenzoy-TRPV1 (803.819 nm^2^), and PINO-TRPV1 (803.486 nm^2^) exhibited lower SASA values compared to dichotomintin-TRPV1 (821.639 nm^2^), irinotecan-TRPV1 (809.598 nm^2^), nodakenin-TRPV1 (809.074 nm^2^), and phellodendrine-TRPV1 (809.502 nm^2^) (Figures 2D,H). This suggests that the former compounds, when bound to TRPV1, may contribute to greater structural stability.

Through an analysis incorporating RMSD, hydrogen bonds, and SASA, upon interaction with TRPV1, the HHDP moiety of PINO was noted to maintain stability within the active pocket, leading to hydrogen bonding formation and reduced SASA of PINO. These findings suggest that PINO forms a tight interaction with TRPV1, thereby enhancing the overall stability of the system.

#### 3.2.4 RMSD of protein

**FIGURE 3:**
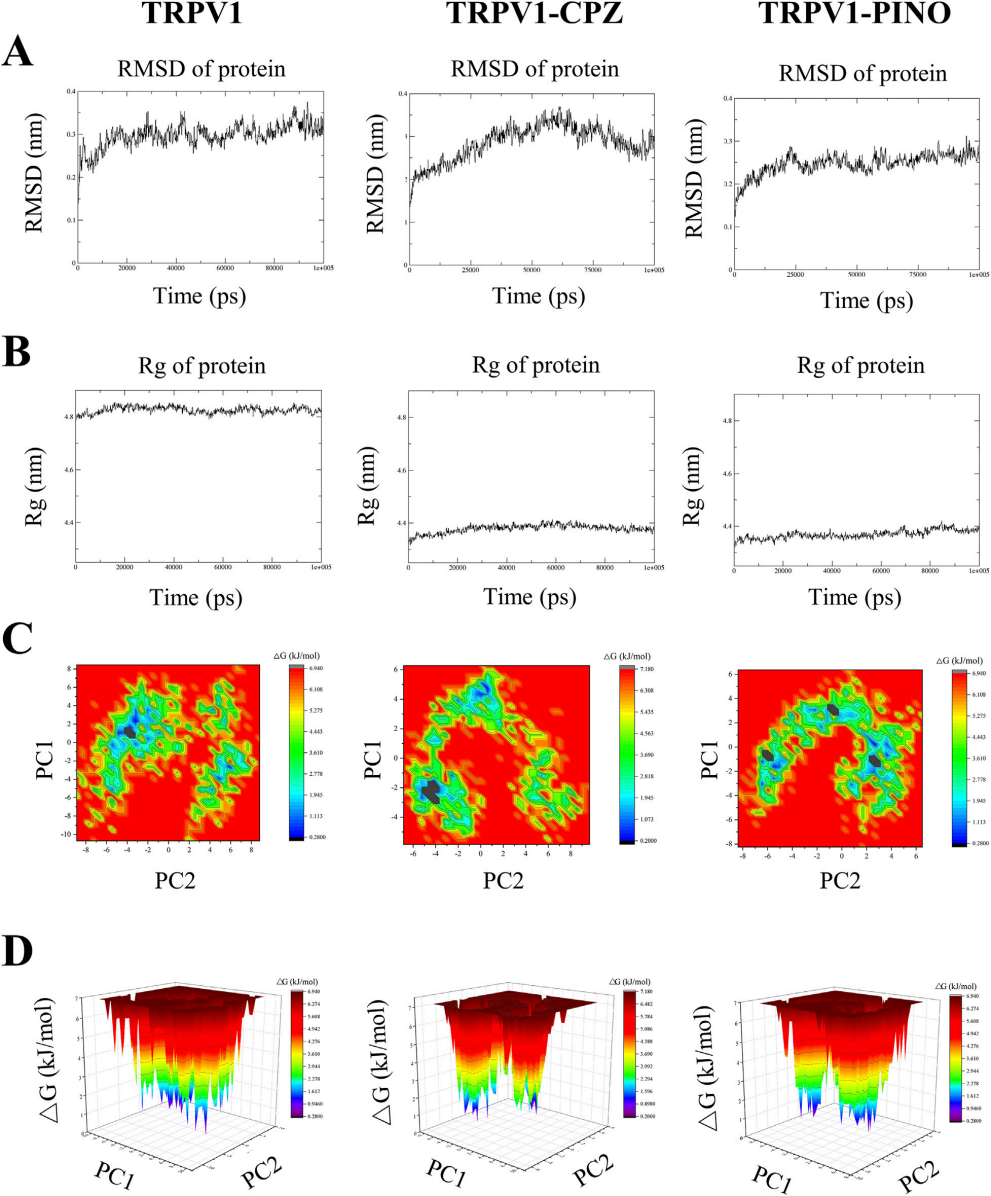
The profile for the MD simulation of unbound TRPV1, TRPV1-CPZ and TRPV1-PINO. The RMSD plot variation of unbound TRPV1 and the TRPV1-ligand complexes **(A)**, Rg plot variation of unbound TRPV1 and the TRPV1-ligand complexes **(B)**, Representation of the two-dimensional projections of the protein conformational changes during simulation using PC1 and PC2 for unbound TRPV1 and the TRPV1-ligand complexes **(C)**, Gibbs free energy landscapes created from MD simulation trajectories of unbound TRPV1 and the TRPV1-ligand complexes **(D)**.

The aforementioned study revealed that PINO and TRPV1 can form a more stable system; however, there was no comparison made with the molecular dynamics of known TRPV1 inhibitors. Therefore, in subsequent research, we selected two known TRPV1 inhibitors, capsazepine (CPZ), to investigate whether PINO exhibits similar molecular dynamics characteristics as these known inhibitors. Figure 3A demonstrates that the most stable complex within the group is the PINO-TRPV1 complex. This stability is attributed to two factors (De Ridder et al., 2021): exhibiting a lower average RMSD value (0.2469 nm) in contrast to unbound TRPV1 (0.2968 nm) and TRPV1-CPZ (0.2846 nm); and (Advisory, 2024) demonstrating minimal fluctuation and stabilization in its spectral graph throughout the entirety of the simulation period.

#### 3.2.5 Radius of gyration (Rg) of protein

The trajectory of the radius of gyration (Rg) of the ligand-protein complexes was examined, offering a metric of the atom(s) mass relative to the center of mass of the molecule (Phd et al., 2018). This metric indicates the structural compactness (Yadav et al., 2013). The average Rg value for unbound TRPV1 was 4.825 nm, whereas TRPV1-CPZ (4.379 nm) and TRPV1-PINO (4.370 nm) exhibited lower average Rg values, suggesting minimal fluctuation and a more compact system. PINO-TRPV1 showed the lowest Rg values of the other two complexes (Figure 3B).

#### 3.2.6 Principal component analysis

Principal component analysis (PCA) is a method employed to condense the dimensionality of a vast dataset to its principal components, revealing noteworthy variations that encapsulate the global motion of the protein and crucial information. The principal components (PCs) obtained during MD simulation are the eigenvector values derived from the covariance matrix, where each PC represents a specific alteration in the protein’s trajectory (Al-Khafaji and Taskin Tok, 2020). A two-dimensional (2D) PCA was performed to analyze the dynamics of ligand-TRPV1 systems, to understand the impact of CPZ and PINO binding on the proteins’ motions. In this study, the first two principal components (PCs), specifically PC1 and PC2, of the Cα atoms were selected to forecast the motions. Figure 3C depicts the projection of the two eigenvectors for the unbound TRPV1, TRPV1-CPZ and TRPV1-PINO complexes. The results indicated that unbound TRPV1 occupied more space, while TRPV1-CPZ and TRPV1-PINO exhibited a more stable cluster compared to unbound TRPV1. The findings suggested that TRPV1-PINO exhibited a stable conformation similar to that of TRPV1-CPZ.

#### 3.2.7 Gibbs free energy landscapes

Utilizing Principal Component Analysis (PCA) to analyze Gibbs free energy landscapes (FELs) provides a comprehensive understanding of the conformational dynamics of a system (Papaleo et al., 2009). In this research, we utilized the initial two principal components as reaction coordinates to differentiate the conformational states of both the unbound TRPV1 and TRPV1-ligand systems (Figure 3D). It can be observed that unbound TRPV1 explores a significant conformational space with two distinct global energy minima. In the case of TRPV1 bound to CPZ, it was observed that the protein undergoes a shift in local minima compared to unbound TRPV1. PINO bound to TRPV1 exhibits similar characteristics to CPZ and results in three local minima. The findings revealed that CPZ and PINO consistently interacted with TRPV1, albeit displaying distinct binding characteristics.

#### 3.2.8 MMPBSA analysis

The MM-PBSA approach was applied to compute the binding free energies of ligand-protein complexes, facilitating the hierarchical classification of binding affinities for these three distinct compounds (Rui et al., 2022). Within this methodology, the binding free energy is dissected into various constituent elements, encompassing intermolecular van der Waals interactions (ΔE_vdW_), electrostatic contributions (ΔE_elec_), nonpolar solvation effects (ΔG_np_), polar solvation free energy (ΔG_pol_), and the configurational entropy (-TΔS). The discrete components contributing to the binding free energies of the three complexes are outlined in Table 2.

**TABLE 2:** Energetic components of the binding energy for three compounds in complex with TRPV1 using MM-PBSA (kJ/mol).

Components	CPZ	PINO
van der Waal energy (ΔE_vdW_)	−155.256	−257.632
Electrostatic energy (ΔE_elec_)	−97.401	−19.076
^a^ΔE_MM_	−252.657	−276.708
Polar solvation energy (ΔG_pol_)	150.791	174.675
Non-polar solvation energy (ΔG_np_)	−26.075	−38.173
Configurational entropy (-TΔS)	47.940	22.510
Binding energy (^b^ΔG_Bind_)	−80.001	−117.696

Note:

^a^ ΔE_MM_ = ΔE_vdW_ + ΔE_elec_.

^b^ ΔG_Bind_ = ΔE_MM_ + ΔG_pol_ + ΔG_np_ − TΔS.

The van der Waals interaction energy (ΔE_vdW_), electrostatic interactions (ΔE_elec_), and nonpolar solvation energy (ΔG_np_) contributed to the formation of complexes with TRPV1 by each ligand. Conversely, the polar solvation-free energy (ΔG_pol_) and the configurational entropy (-TΔS) worked against the binding process (Rui et al., 2022). The van der Waal energy is the main force driving TRPV1-ligands complexation.

The findings indicated that PINO exhibited the most favorable binding free energy among the three compounds, suggesting its potential to establish the most stable complex with TRPV1, while CPZ ranked third. Collectively, these results suggest that PINO is the most potent binder among the three compounds.

### 3.3 Calcium imaging

**FIGURE 4:**
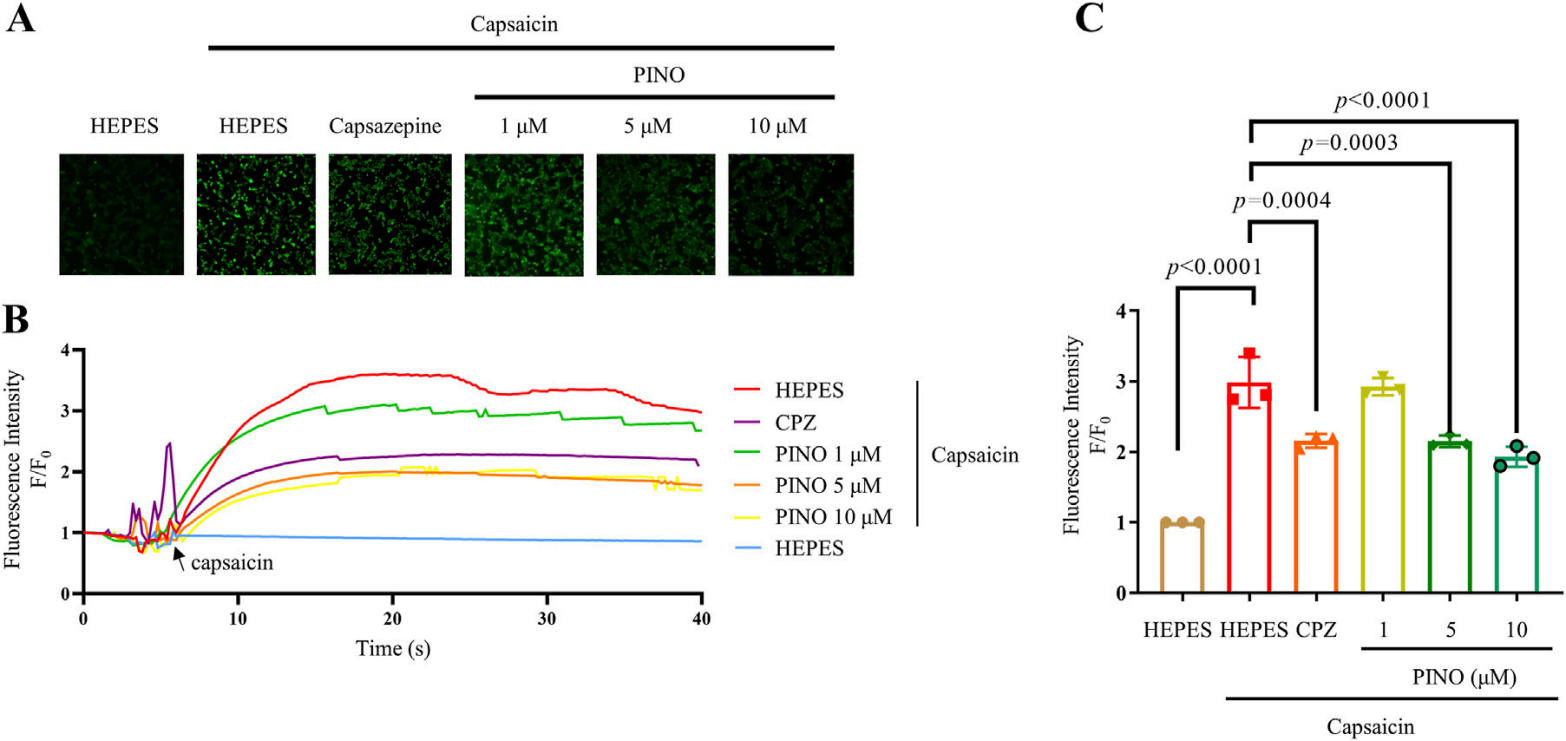
Calcium imaging of different concentrations ranging from 1 to 10 μM of PINO on HEK293 cells expressing human TRPV1. Representative images showing intracellular calcium fluorescence intensity of HEK293-hTRPV1 cells (n = 3) **(A)**. Representative time-dependent response of calcium fluorescence intensity of PINO in HEK293-hTRPV1 cells **(B)**. Normalized relative calcium fluorescence intensity of PINO in HEK293-hTRPV1 cells **(C)**. Data are expressed as mean ± SD, Data were standardized to the HEPES control group. Statistical analysis by one-way ANOVA with Bonferroni’s *post hoc* test.

The fluorescent calcium imaging was conducted on HEK293-hTRPV1 cells (see Figure 4). *In vitro* studies employed calcium imaging techniques to investigate calcium flow through the channel, utilizing transfected cells such as HEK293 cells expressing either hTRPV1 or rTRPV1 (Abbas, 2020). Stimulation with CPZ decreased the calcium fluorescence while varying concentrations of PINO also reduced the calcium fluorescence. Significantly, 5 μM and 10 μM of PINO demonstrated a notable impact on TRPV1, with its fluorescence intensity peak lower than that of CPZ (10 μM). The results indicated that PINO exhibited a stronger inhibitory effect on the TRPV1 channel compared to CPZ. Other 9 compounds including asarinin, beta-carotene, columbianadin, corylin, 3,29-dibenzoyl rarounitriol, dichotomitin, irinotecan, nodakenin, phellodendrine did not inhibit the Ca^2+^ influx (Supplementary Figure S3). In addition, PINO alone does not activate TRPV1-mediated calcium signaling, consistent with its proposed role as a modulator rather than a direct agonist.

### 3.4 Pain behavioral tests

#### 3.4.1 Acetic acid-induced abdominal stretching

**FIGURE 5:**
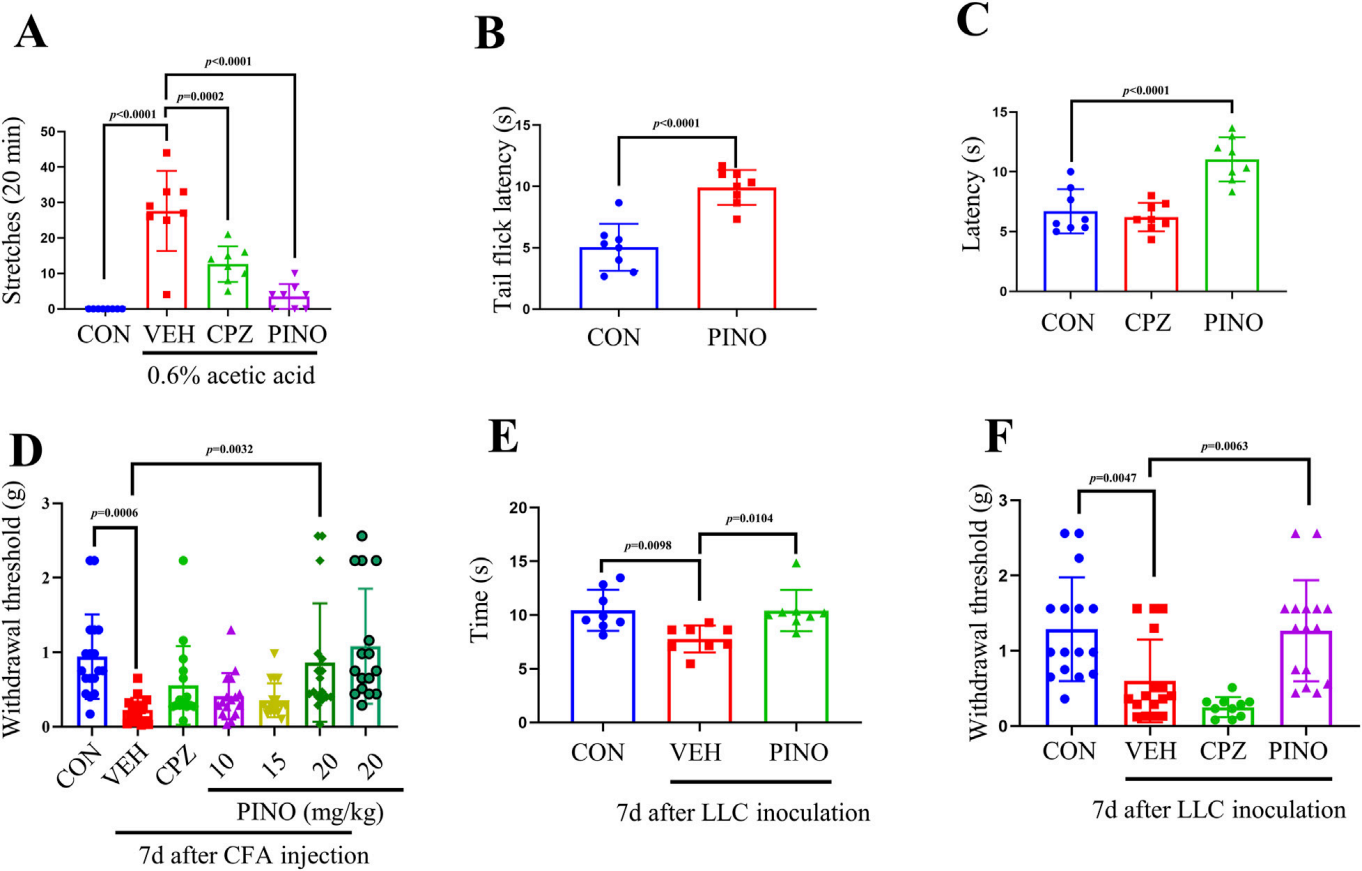
PINO analgesic effect was determined by different animal tests. Total number of writhing episodes within 20 min after 0.6% acetic acid injection (n = 8) **(A)**. Thermal sensitivity was assessed by hot water tail-flick test (n = 8) **(B)** and hot plate assay (n = 8) **(C)**. The mechanical withdrawal threshold was assessed using the von Frey test in the CFA-induced inflammatory pain model (n = 16–18) **(D)**. Thermal and mechanical sensitivities in bone cancer pain induced by LLC inoculation were evaluated using the hot plate test (n = 8) **(E)** and von Frey test (n = 10–16) **(F)**. The dose of CPZ was 20 mg/kg (administered intraperitoneally). Data are presented as mean ± SD. Statistical analysis by unpaired 2-tailed Student t-test in **(B)** or one-way ANOVA with Bonferroni’s *post hoc* test in **(A)**, **(C)**, **(D)**, **(E)** and **(F)**.

Following intraperitoneal injection of 0.6% acetic acid in saline, mice exhibited trunk curving and limb extension, indicative of a stretch response. Normally, the onset of stretching was observed between 2 and 5 min post-acetic acid administration in control mice (Naidu et al., 2009). Pre-administration of CPZ at a dose of 20 mg/kg notably decreased the occurrence of acetic acid-induced abdominal stretches in mice compared to the vehicle-treated group. Interestingly, PINO at a dose of 20 mg/kg exhibited a more pronounced reduction in the number of stretches compared to CPZ (refer to Figure 5A).

#### 3.4.2 Hot water tail-flick test

The heat pain sensitivity in mice can be assessed using the hot water tail-flick method (Tian et al., 2016). Treatment with PINO at a dose of 20 mg/kg prolonged the latency of the tail-flick response to 50°C hot water in mice when compared to the control group (refer to Figure 5B).

#### 3.4.3 Hot plate test

The tail-flick method primarily evaluates pain sensitivity at the spinal level, whereas the hot plate test predominantly assesses pain responses and analgesia mediated by supraspinal mechanisms (Gomeza et al., 1999).

In the hot plate test, the administration of CPZ at a dosage of 20 mg/kg did not affect the thermal threshold. Additionally, PINO (20 mg/kg) exhibited a significant analgesic effect compared to the TRPV1 inhibitor CPZ, suggesting potential neuronal hyperactivity (Figure 5C) (Wang Z. et al., 2020).

#### 3.4.4 CFA-induced inflammatory pain

The initiation/acute phase of CFA-induced inflammatory pain typically endures for around 2 days, following which the pain transitions into a persistent state lasting for several weeks (Xiang et al., 2019). All mice injected with CFA exhibited both mechanical allodynia and thermal hyperalgesia, with the paw withdrawal threshold reaching its lowest value on day 7 (Hua et al., 2022). The paw withdrawal threshold in the vehicle group was notably lower compared to the control group, as shown in Figure 5D. A single intraperitoneal administration of CPZ (20 mg/kg) on day 7 post-CFA injection did not significantly alleviate mechanical allodynia and thermal hyperalgesia. However, 20 mg/kg of PINO can significantly increase the paw withdrawal threshold compared with the vehicle group. In addition, a single intraperitoneal injection of PINO (20 mg/kg) without CFA injection did not alter the paw withdrawal threshold compared with the control group.

#### 3.4.5 Bone cancer pain

In mice, the intra-femoral inoculation of Lewis lung carcinoma (LLC) cells mimics the characteristics of bone cancer pain (Yang et al., 2018). In the hot plate test, the vehicle group exhibited a significant decrease in latency after LLC inoculation, whereas 20 mg/kg of PINO induced notable antinociception on the 56°C hot plate (Figure 5E). Concerning mechanical sensitivity, following LLC inoculation, the paw withdrawal threshold declined from 1.29 ± 0.67 g before cell inoculation (baseline) to 0.60 ± 0.53 g on day 7 in von Frey tests (Figure 5F). However, no significant alteration in pain perception was noted in the CPZ group compared to the vehicle group. In contrast, after the administration of PINO at a dose of 20 mg/kg, a significant enhancement in the paw withdrawal threshold to 1.27 ± 0.65 g was observed. Our results suggest that PINO alleviates bone cancer pain.

### 3.5 The effect of PINO on CFA-induced inflammatory pain in plasma and dorsal root ganglion (DRG) of mice

**FIGURE 6:**
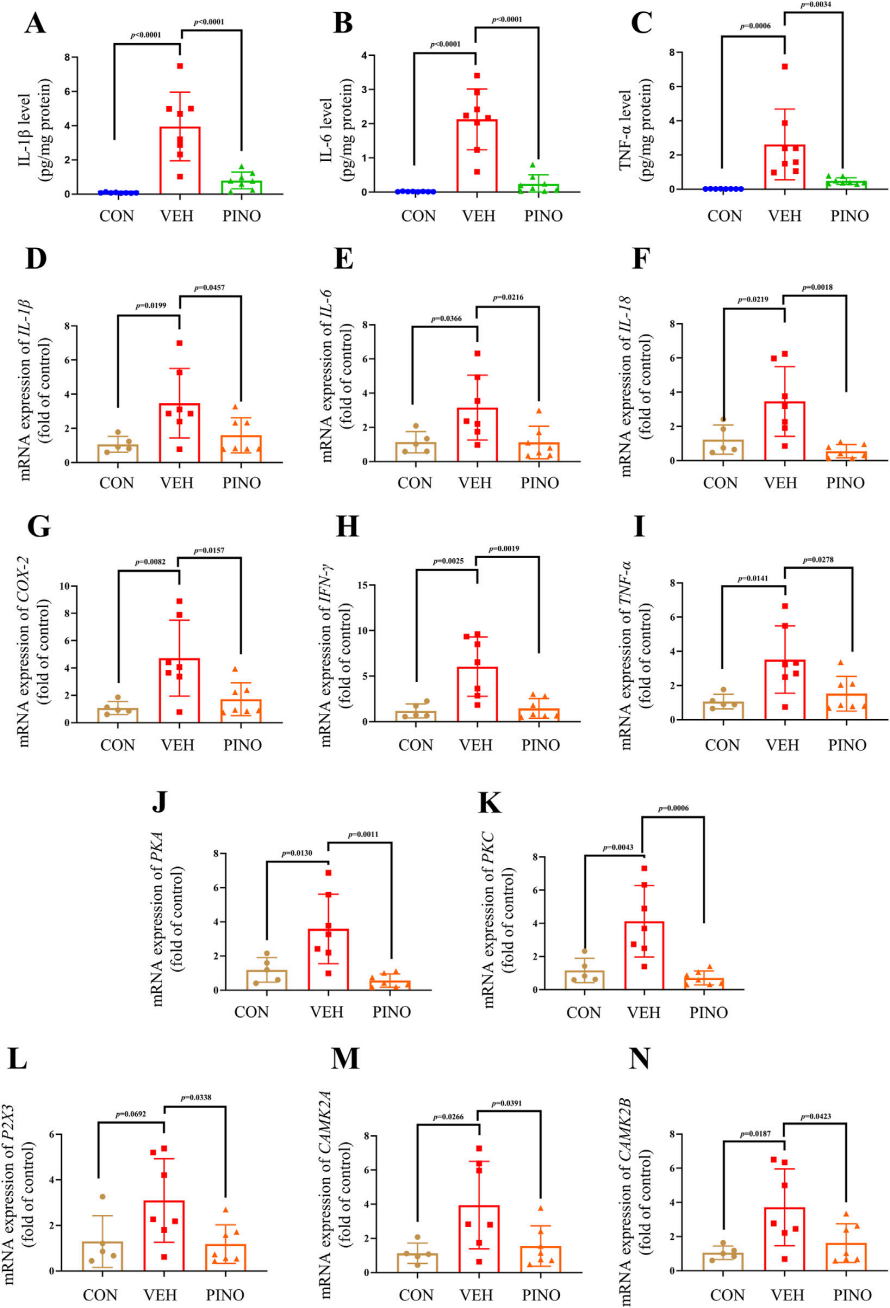
The effect of PINO on CFA-induced inflammatory pain in the plasma and the DRG of mice. Plasma analysis: PINO effects on the secretion of IL-1β, IL-6, and TNF-α (n = 8). **(A–C)** (n = 8). DRG analysis: PINO effects on mRNA expression of *IL-1β*, *IL-6*, *IL-18*, *COX-2*, *IFN-γ*, *TNF-α*, *PKA*, *PKC*, *P2X3*, *CAMK2A* and *CAMK2B* **(D–N)** (n = 5–7). Data are presented as mean ± SD. Statistical analysis by one-way ANOVA with Bonferroni’s *post hoc* test.

To clarify the potential mechanism of PINO in CFA-induced inflammatory pain, an analysis was conducted on the secretion in plasma, mRNA expression of inflammatory cytokines, and Ca^2+^-related genes in the DRG of mice. The findings indicated that the administration of CFA increased the secretion of distinct inflammatory cytokines such as IL-1β, IL-6, and TNF-α (see Figures 6A–C). Additionally, the administration of 20 mg/kg PINO resulted in a reduction in the secretion of these cytokines. Furthermore, regarding mRNA expression, the injection of CFA elevated the mRNA levels of *IL-1β*, *IL-6*, *IL-18*, *COX-2*, *IFN-γ*, and *TNF-α*, along with genes associated with the Ca^2+^ signaling pathway including *PKA*, *PKC*, *P2X3*, *CAMK2A*, and *CAMK2B* (Figures 6D–N). On the contrary, the application of PINO led to a reduction in the expression of both inflammatory genes and Ca^2+^-related genes. The results suggested that the analgesic effect of PINO may be attributed to its targeting of TRPV1, subsequently exerting an anti-inflammatory effect.

### 3.6 The anti-inflammatory effect of PINO on TNF-α-induced RAW264.7 cells

**FIGURE 7:**
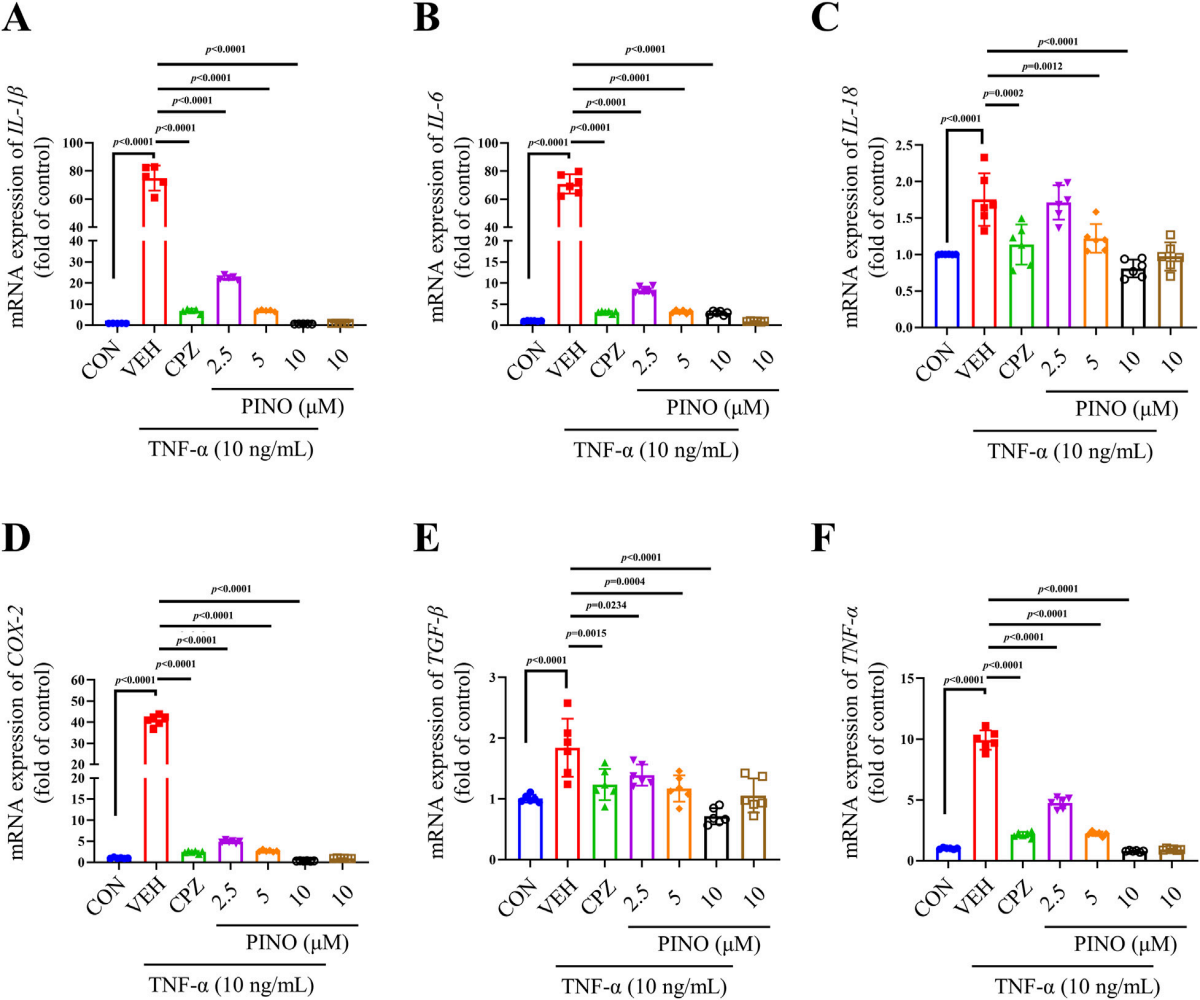
The anti-inflammatory effects of PINO on the mRNA expression of *IL-1β*, *IL-6*, *IL-18*, *COX-2*, *TGF-β*, and *TNF-α* **(A–F)** in TNF-α-induced RAW264.7 cells (n = 6). Data are presented as mean ± SD. Statistical analysis by one-way ANOVA with Bonferroni’s *post hoc* test.

The toxicity evaluation of PINO in RAW264.7 cells indicated no toxic effects at concentrations up to 100 μM (Supplementary Figure S4). Considering the important role of TNF-α in the progression of inflammation and our previous observations suggest that CFA can stimulate TNF-α production, resulting in inflammation. The research was focused on examining the anti-inflammatory effects of PINO on TNF-α-induced RAW264.7 cells (refer to Figure 7). Exposure to 10 ng/mL TNF-α led to an upregulation in the mRNA expression of *IL-1β*, *IL-6*, *IL-18*, *COX-2*, *TGF-β*, and *TNF-α*. Subsequent coculture with CPZ resulted in a significant decrease in the expression of all these genes. Furthermore, the varying concentrations of PINO demonstrated the ability to reduce the expression of these genes, indicating that PINO could counteract the upregulation of inflammatory genes induced by TNF-α.

### 3.7 PINO‐mediated anti-inflammatory effect is dependent of NF-κB/MAPK signaling pathways

**FIGURE 8:**
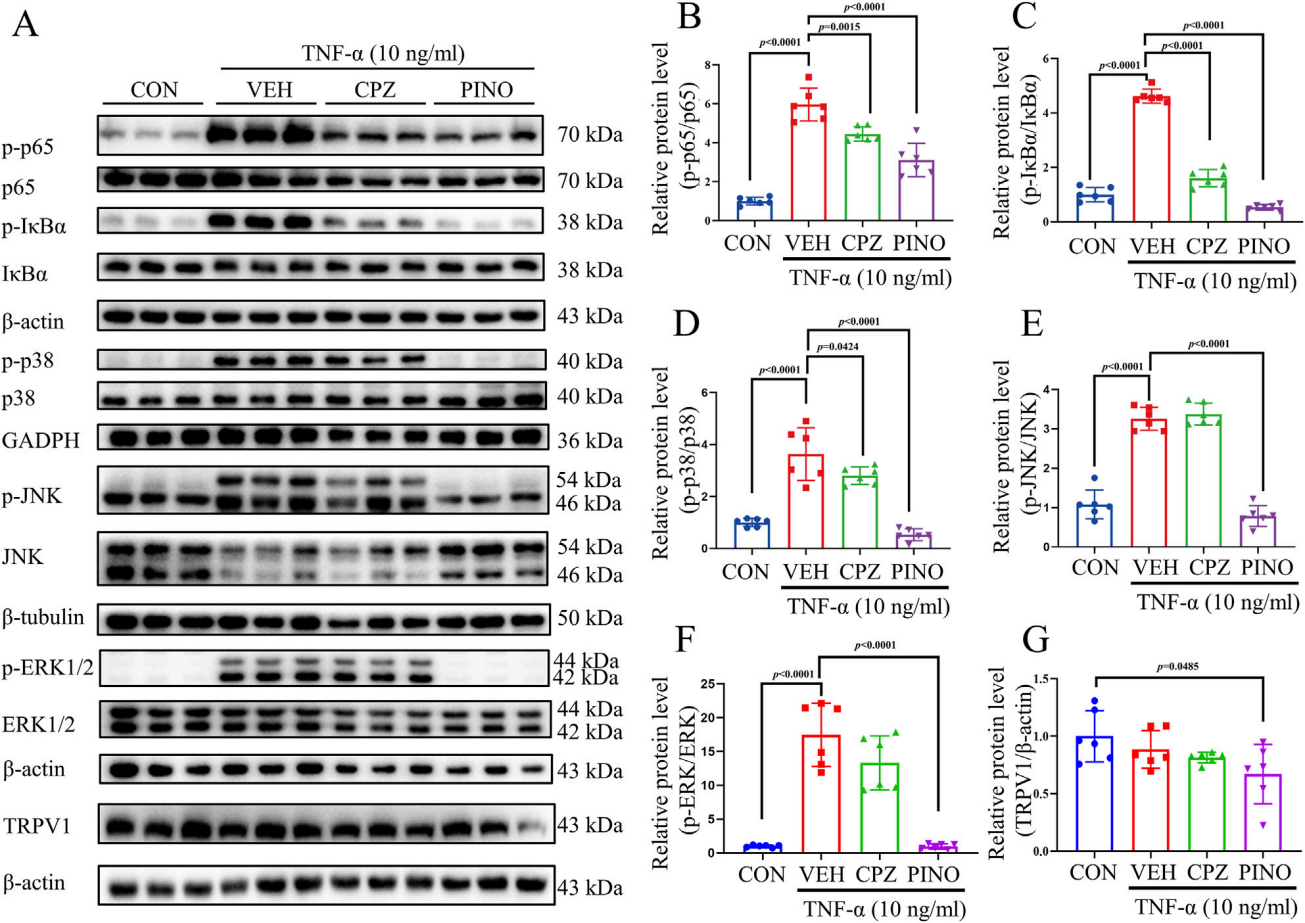
The effect of PINO on NF-κB and MAPK pathways. RAW264.7 cells were treated with CPZ (10 μM) or PINO (10 μM) for 1 h, and whole-cell lysates were prepared and immunoblotted using p-p65, p65, p-IκBα, IκBα, p-p38, p38, p-JNK, JNK, p-ERK1/2, ERK1/2, TRPV1, β-actin, GAPDH, and β-tubulin antibody **(A)**. Fold change was determined after normalization with β-actin, GAPDH, or β-tubulin, with the right panels illustrating the fold change relative to the control: p-p65/p65 **(B)**, p-IκBα/IκBα **(C)**, p-p38/p38 **(D)**, p-JNK/JNK **(E)**, p-ERK1/2/ERK1/2 **(F)** and TRPV1/β-actin **(G)** (n = 6). Data are presented as mean ± SD. Statistical analysis by one-way ANOVA with Bonferroni’s *post hoc* test.

TNF-α can induce inflammation by activating the NF-κB/MAPK signaling pathway (Wu et al., 2022). In previous research, inhibition of NF-κB has been shown to alleviate CFA-induced mechanical and thermal pain in rats (Hartung et al., 2015). This study explores the anti-inflammatory effects of PINO on RAW267.4 cells via the NF-κB/MAPK signaling pathways. The results demonstrated that TNF-α induced the phosphorylation of p65, IκBα, p38, JNK, and ERK1/2 (Figure 8). Conversely, treatment with 10 μM of CPZ decreased the phosphorylation of p65 and IκBα, while it did not affect the increase in p38, JNK, and ERK1/2 induced by TNF-α. Subsequently, treatment with 10 μM of PINO not only reduced the phosphorylation of p65 and IκBα but also decreased the phosphorylation of p38, JNK, and ERK1/2. These findings suggest that the anti-inflammatory effects of PINO are associated with the inhibition of NF-κB/MAPK signaling pathways. We next investigated whether PINO modulates TRPV1 expression in macrophages. The result showed that revealed constitutive expression of TRPV1 in untreated RAW264.7 cells, with PINO treatment resulting in a modest but consistent reduction in TRPV1 protein levels compared to control group.

To determine whether PINO’s effects are mediated through TRPV1, we employed a pharmacological intervention using the TRPV1 agonist capsaicin. Notably, co-treatment with capsaicin substantially attenuated PINO’s suppression of NF-κB activation and phosphorylation of p38 and ERK (Supplementary Figure S5), suggesting competitive interaction at TRPV1 binding sites or downstream signaling crosstalk.

## 4 Discussion

This study aims to discover a novel painkiller from natural products through virtual screening. In this study, a new TRPV1 antagonist was identified and designated as pinocembrin 7-O-[3″-O-galloyl-4″,6″-hexahydroxydiphenoyl]-β-D-glucoside (PINO), was first extracted from the entire plant of *Penthorum chinense P* (Wang et al., 2006). Previous studies have shown that PINO displays a moderate inhibitory effect on α-amylase activity, with an IC_50_ value of 0.03 μmol/mL (Huang et al., 2015). Moreover, PINO demonstrated efficacy in alleviating various forms of pain, including acetic acid-induced, thermal-induced, CFA-induced inflammatory pain, and LLC cell-induced bone cancer pain *in vivo*. Furthermore, it was observed that PINO could diminish the secretion or mRNA expression of inflammatory cytokines in the plasma or DRG associated with CFA-induced inflammatory pain. Additionally, PINO exhibited an anti-inflammatory effect by regulating the NF-κB and MAPK signaling pathways in RAW264.7 cells. Consequently, we posit that PINO merits consideration as a promising analgesic agent with significant potential applications.

The TRPV1 channel belongs to the vanilloid subfamily of TRP channels, which was first discovered as a heat-sensing receptor in the pain pathway. Numerous researches have demonstrated that inhibition of TRPV1 leads to reducing the Ca^2+^ influx and subsequently alleviating pain. Thus, the antagonist of TRPV1 is considered a promising analgesia agent (Deng et al., 2018). With the notable side effect profiles associated with steroidal and NSAID medications, there is a growing focus on natural compounds, including dietary supplements and herbal remedies. These have a historical usage spanning century in alleviating pain and inflammation (Maroon et al., 2010).

To expedite the drug discovery process, structure-based virtual screening was employed in this study. Ten compounds were selected and subjected to molecular dynamics simulation. In addition, a greater number of hydrogen bonds between the ligand and protein can further reduce the binding energy of the protein-ligand complex (Singh et al., 2021). Dichotomitin, nodakenin, and PINO exhibited the ability to engage in the highest number of hydrogen bonds with TRPV1 over a 100 ns simulation period compared to other compounds. While corylin, dibenzoyl, and phellodendrine demonstrated a relatively elevated average number of hydrogen bonds with TRPV1, their inability to sustain continuous formation during molecular dynamics simulations suggest a diminished binding affinity to TRPV1 for these compounds. Interestingly, the PINO-TRPV1 complex displayed a decreasing trend in the SASA during the simulation, indicating a more compact configuration. On the contrary, dichotomitin and nodakenin may demonstrate a higher SASA value than PINO. As a result, PINO was chosen for further scrutiny.

Next, we employed established TRPV1 inhibitors CPZ for comparative analysis, aiming to elucidate the PINO’s binding mode with TRPV1 compared to these inhibitors. Regarding the RMSD values of the protein, PINO displayed comparable fluctuations when interacting with TRPV1, suggesting that their binding preserves the stability of TRPV1. In contrast, CPZ exhibited significant fluctuations over a 50 ns simulation period. The Rg value suggested that CPZ and PINO contribute to enhanced structural stability of TRPV1 in comparison to the unbound state. The results suggested that TRPV1-CPZ and TRPV1-PINO exhibit comparable features while forming distinct local minima compared to unbound TRPV1 in PCA and Gibbs’s free energy, signifying conformational alterations in TRPV1.

Subsequently, utilizing the acquired trajectories and the MM/PBSA method, we computed a revised binding free energy, which theoretically offers greater computational robustness compared to the score derived from molecular docking (García-Ariza et al., 2022). In the binding energy assessment conducted through MM-PBSA, the analysis revealed that van der Waals energy played a predominant role in mediating the binding interactions between TRPV1 and the three compounds. Moreover, the binding energy evaluation indicated that the TRPV1-PINO complex displayed a significantly more stable conformation compared to the TRPV1-CPZ complex, highlighting its superior potential for inhibiting TRPV1. This inhibitory effect of PINO was validated through calcium imaging experiments conducted in HEK293 cells overexpressing hTRPV1.

Given PINO’s ability to attenuate calcium influx by inhibiting TRPV1, it has been identified as a promising candidate for analgesic applications. Therefore, it is plausible to hypothesize that PINO may modulate various pain modalities (such as acid, heat, and mechanical stimuli) in diverse pain models. Sex differences in pain perception are indeed critical, particularly as estrogen has been shown to upregulate NMDA, TRPA1, and TRPV1 receptors, thereby potentiating pain signaling (Athnaiel et al., 2023). To isolate the analgesic effect of PINO without interference from estrogen fluctuations, we selected male mice for this initial study. The results indicated that PINO reduced the writhing response caused by acetic acid, raised the thermal pain threshold in both the hot water tail-flick test and hot plate assay, and improved the mechanical pain threshold in CFA-induced inflammatory pain, as evaluated by the von Frey test. This finding demonstrated that PINO exhibits efficacy against acute inflammatory, acute thermal, chronic inflammatory pain, and bone cancer pain (Wu et al., 2019).

The administration of CFA led to the establishment of enduring inflammatory pain. The TRPV1 expression exhibited sustained elevation in both the dorsal root ganglion (DRG) and the spinal cord (SC) of mice for 28 days after the onset of inflammatory pain (Yen et al., 2019). IL-1β, IL-6, and TNF-α represent crucial proinflammatory cytokines (Gao et al., 2020). IL-18, a proinflammatory cytokine, exerts regulatory effects on inflammation and immune responses (Xu et al., 2021). COX-2, an inducible isoform of cyclooxygenase, assumes a pivotal role in inflammation by generating PGE_2_, which is also implicated in inflammatory processes (Lim et al., 2016). IFN-γ triggers macrophage activation and plays a pivotal role in fostering inflammation (Yamaguchi et al., 2022). TRPV1 harbors multiple phosphorylation sites for protein kinase A (PKA) and protein kinase C (PKC), thereby modulating channel sensitization and desensitization (Ho et al., 2014). In a preclinical investigation, unidirectional cross-desensitization between P2X purinoceptors and vanilloid receptors was observed in mature rat DRG neurons. This suggests a possible physiological interaction between P2X3 and TRPV1 (Yu et al., 2021). The activation of CaMK2A and CaMK2B is induced by calcium (Ca^2+^) influx, leading to subsequent calcium-calmodulin (Ca^2+^-CaM) binding (Yasuda et al., 2022). Our results indicated that PINO not only reduced the secretion of inflammatory cytokines but also inhibited the mRNA expression of inflammatory genes and their related calcium ion-associated genes. The mechanism elucidated that PINO exerts its analgesic effects through the modulation of the TRPV1 channel, thereby inhibiting inflammation.

Subsequently, the anti-inflammatory properties and underlying mechanism of action of PINO were assessed in RAW264.7 cells. TNF-α can trigger the phosphorylation of IκBα and p65, leading to the enhanced translocation of p65 from the cytoplasm to the nucleus (Van Quickelberghe et al., 2018). The binding of TNF-α to cell surface receptors initiates diverse signal transduction pathways that engage three types of mitogen-activated protein (MAP) kinases: extracellular-signal-regulated kinases (ERKs), cJun NH2-terminal kinases (JNKs), and p38 MAP kinases (Sabio and Davis, 2014). Activation of NF-κB and MAPK signaling pathways elicits a secondary response by enhancing the expression of various inflammatory cytokines, such as TNF-α (Sabio and Davis, 2014). Our study results showed that PINO decreased the mRNA expression of inflammatory genes and inhibited the phosphorylation of p65, IκBα, p38, JNK, and ERK1/2. These findings suggest that PINO, acting on TRPV1, suppressed the NF-κB and MAPK signaling pathways, resulting in inflammation suppression and a significant analgesic effect *in vivo*. The observed downregulation of TRPV1 following PINO treatment suggested that PINO may modulate the expression of TRPV1. In addition, the diminished inhibitory efficacy of PINO when co-administered with capsaicin provides functional evidence for TRPV1 engagement. This agonist-competition pattern implied that PINO may act as an antagonist at TRPV1 or modulate TRPV1-mediated downstream signaling crosstalk with NF-κB and MAPK pathways. While further structural studies were needed to elucidate precise binding mechanisms, these findings position TRPV1 as a key mediator of PINO’s immunomodulatory effects.

Nonetheless, this study has some limitations. The distinct impact of PINO on TRPV1 inhibition in comparison to CPZ has been clarified via principal component analysis (PCA) and analysis of the Gibbs free energy landscape. The mechanism by which PINO influences the closure of TRPV1 channels and its specific impact on the corresponding amino acid alterations remains yet to be elucidated, necessitating additional experimental inquiry. Moreover, PINO exhibited no cytotoxicity *in vitro* (refer to Supplementary Figure S4) and did not significantly perturb mice body temperature (ΔT < 0.5°C), in contrast to other TRPV1 inhibitors (Supplementary Figure S6). Furthermore, PINO exhibits promising therapeutic efficacy in the management of bone cancer pain, prompting additional investigation into its implications in the field of oncological pain management.

In this investigation, virtual screening methodologies were employed to uncover the potential inhibitory properties of natural compounds on TRPV1. Among the ten compounds selectively screened, PINO emerged as a noteworthy candidate. Subsequent molecular dynamics simulations illustrated that PINO shares similarities with established TRPV1 inhibitors such as CPZ. Calcium imaging experiments confirmed its capacity to impede the influx of Ca^2+^ through TRPV1 channels. Notably, PINO displayed notable analgesic effects in both chronic and acute pain conditions, showcasing particularly favorable outcomes in the domain of bone cancer pain. Moreover, it was observed that PINO exerts a pronounced inhibitory influence on inflammatory mediators, attributable to its modulation of the NF-κB and MAPK signaling pathways. These findings suggested that PINO may serve as a potent analgesic agent capable of modulating and mitigating the progression of acute, chronic, and bone cancer-related pain.

## Data Availability

The original contributions presented in the study are included in the article/Supplementary Material, further inquiries can be directed to the corresponding authors.

## Abbreviations

ATCC, American Type Culture Collection; CFA, Complete Freund’s Adjuvant; COX-2, cyclooxygenase 2; CPZ, Capsazepine; DRG, dorsal root ganglia; ELISA, enzyme-linked immunosorbent assay; FBS, Fetal bovine serum; FEL, free energy landscape; GAPDH, glyceraldehyde 3-phosphate dehydrogenase; HEK293, human embryonic kidney cells; IL-1β, interleukin-1 beta; IL-6, interleukin-6; LLC, Lewis lung carcinoma; MAPK, Mitogen-Activated Protein Kinases; MD, Molecular dynamics; NF-κB, nuclear-factor kappa-light-chain enhancer of activated B cells; NO, nitric oxide; NPT, isothermal-isobaric ensemble; NVT, canonical ensemble; PBS, phosphate-buffered saline; PCA, principal component analysis; PDB, Protein Data Bank; PINO, Pinocembrin-7-o-3-o-galloyl-4-6-hexahydroxydiphenoyl-beta-d-glucoside; PMSF, protease inhibitor phenylmethanesulfonyl fluoride; PVDF, Polyvinylidene difluoride; Rg, radius of gyration; RIPA, radioimmunoprecipitation assay lysis buffer; RMSD, root mean square deviation; SASA, solvent-accessible surface area; SEM, Standard Error of the Mean; SD, standard deviation; SPF, specific pathogen-free; TNF-α, tumor necrosis factor-α; TRPV1, transient receptor potential cation channel subfamily V member 1.
