# Supplementary material for: Identification and evaluation of a pinocembrin analog as a TRPV1 inhibitor with analgesic properties in murine pain models
Source: Front Pharmacol. 2025 Jun 10;16:1585181. doi: 10.3389/fphar.2025.1585181 (PMC12185462; doi:10.3389/fphar.2025.1585181)
Supplement: Supplementary file 1 [file Supplementaryfile1.docx]

Supplementary Material

# Supplementary Figures and Tables

## Supplementary Figures


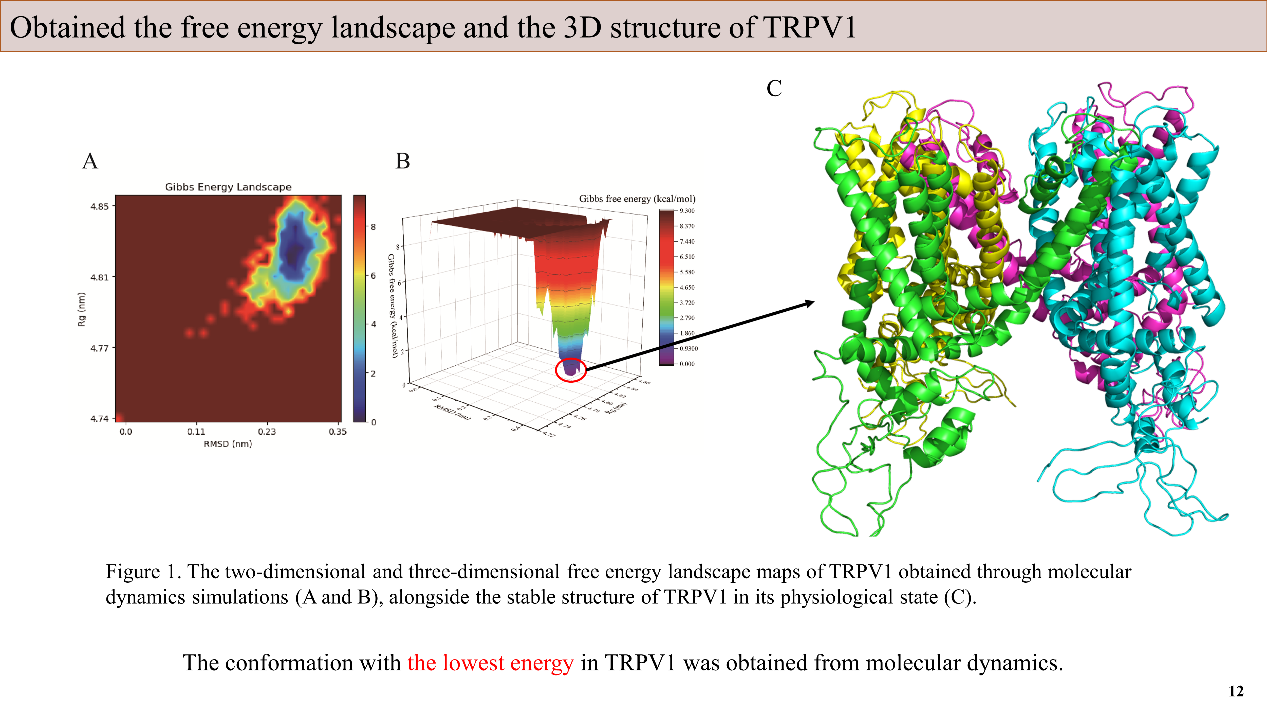


**Supplementary Figure 1.** The two-dimensional representation of Free energy landscape (FEL) of the TRPV1 conformation ensembles using Rg (in nm) versus RMSD (in nm) which was produced by the molecular dynamics simulations **(A)**. The three-dimensional representation of the relative Gibbs free energy of TRPV1. The blue colour represents highest occurrence, green and yellow represent low and red represent lowest occurrence **(B)**. The free energy surface was plotted using Origin. TRPV1 conformation from the highest occurrence represents the lowest Gibbs free energy **(C)**.


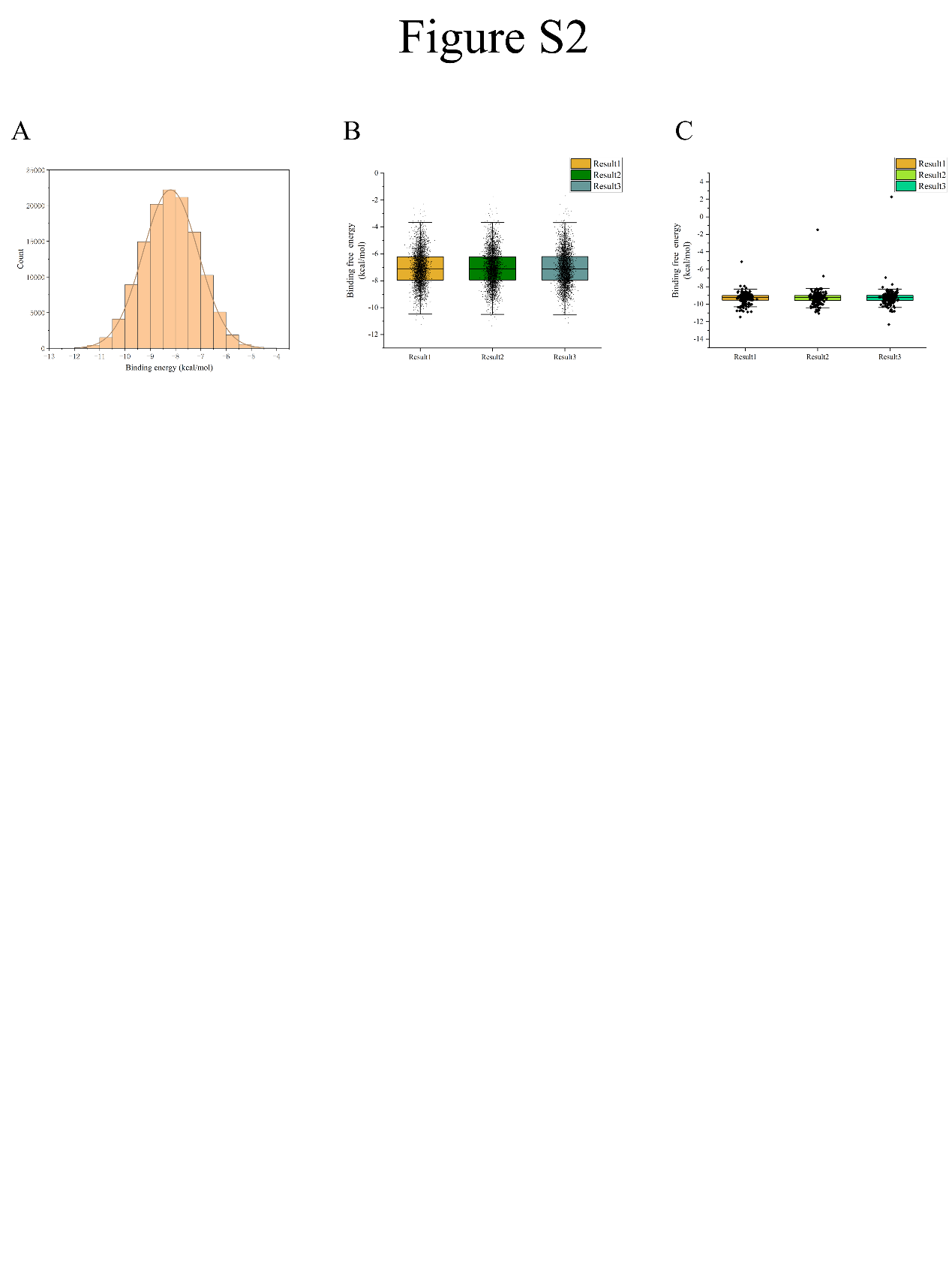


**Supplementary Figure 2.** The virtual screening results. The fast docking of the 127719 natural product library **(A)**. The binding free energy from the triple semi-flexible docking of 4243 selected compounds **(B)**. The binding free energy from the triple flexible docking of 186 selected compounds **(C)**.


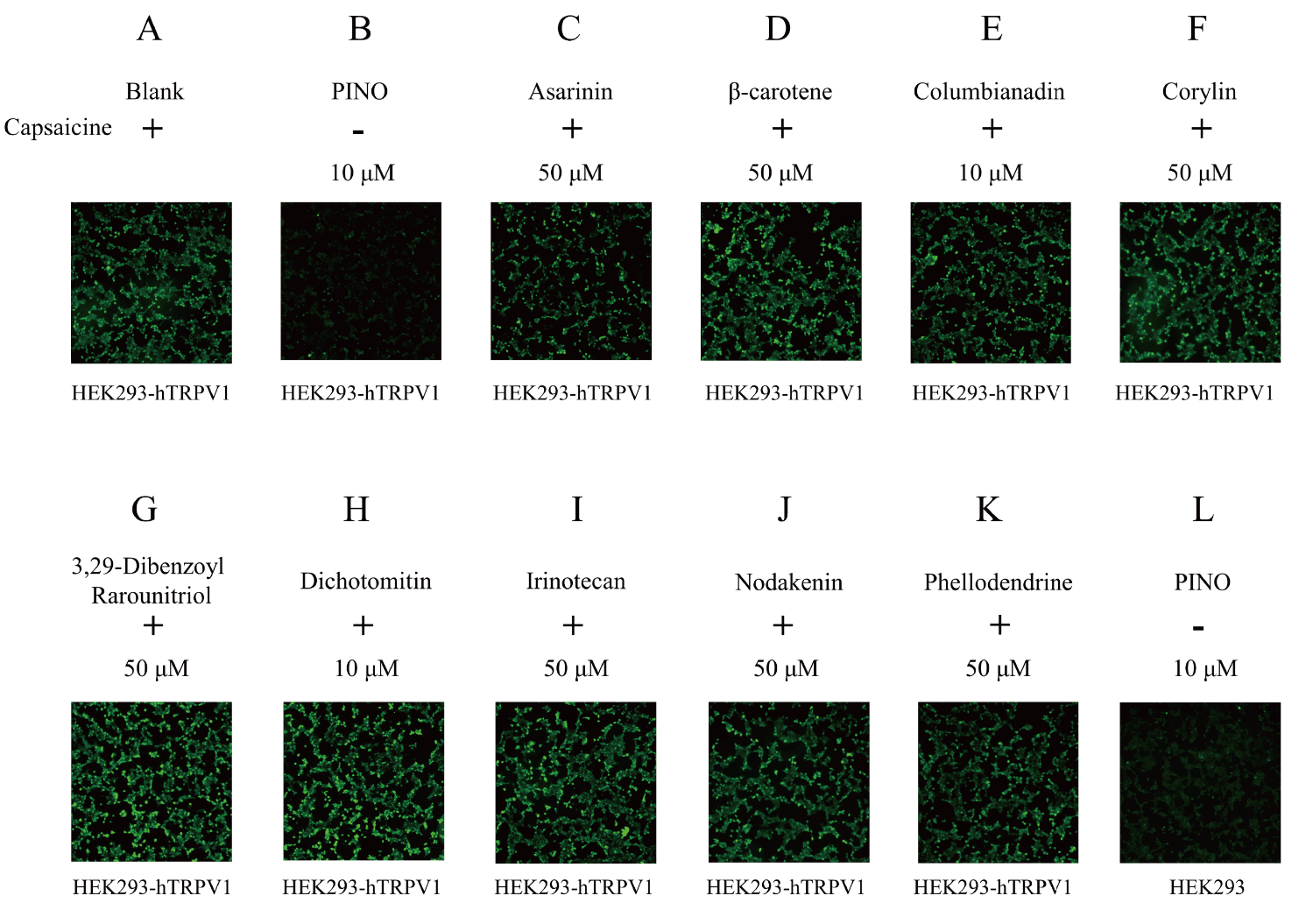


**Supplementary Figure 3.** The effect of Blank, PINO, asarinin, β-carotene, columbianadin, corylin, 3,29-dibenzoyl rarounitriol, dichotomitin, irinotecan, nodakenin and phellodendrine with capsaicine in HEK293-hTRPV1 cells (A-K); The effect of PINO in HEK293 cells (L).


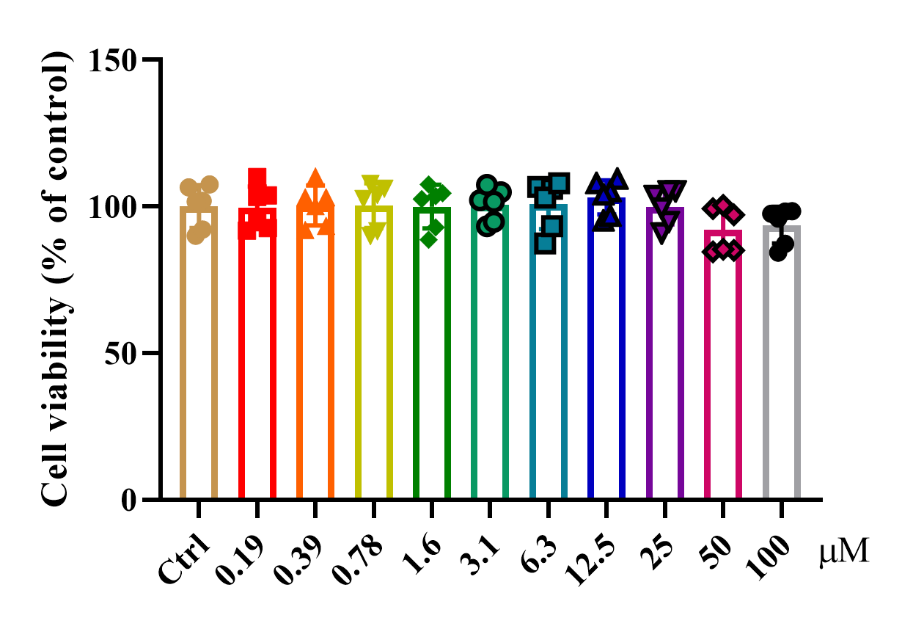


**Supplementary Figure 4.** The cell viability of PINO on RAW264.7 cells.


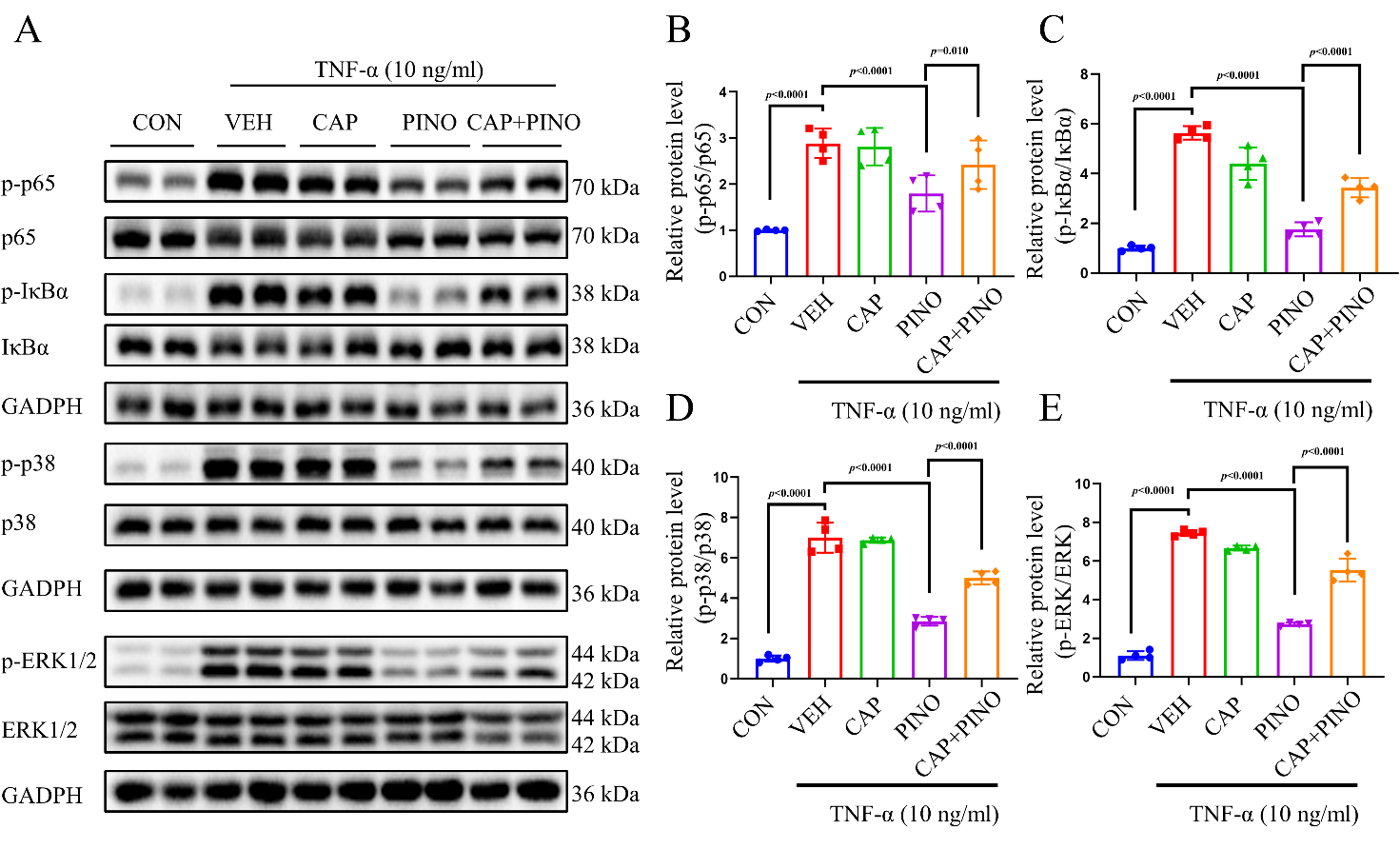


**Supplementary Figure 5.** The effect of PINO and capsaicine (CAP) on NF-κB and MAPK pathways. RAW264.7 cells were treated with CAP (10 μM), PINO (10 μM) alone or CAP (10 μM) combine with PINO (10 μM) for 1 h, and whole-cell lysates were prepared and immunoblotted using p-p65, p65, p-IκBα, IκBα, p-p38, p38, p-ERK1/2, ERK1/2, and GAPDH antibody (A). Fold change was determined after normalization with GAPDH with the right panels illustrating the fold change relative to the control: p-p65/p65 (B), p-IκBα/IκBα (C), p-p38/p38 (D), p-ERK1/2/ERK1/2 (E) and TRPV1/β-actin (n=4). Data are presented as mean ± SD. Comparisons of parameters were performed with 2-way ANOVA followed by the Tukey honestly significant difference test for multiple comparisons.


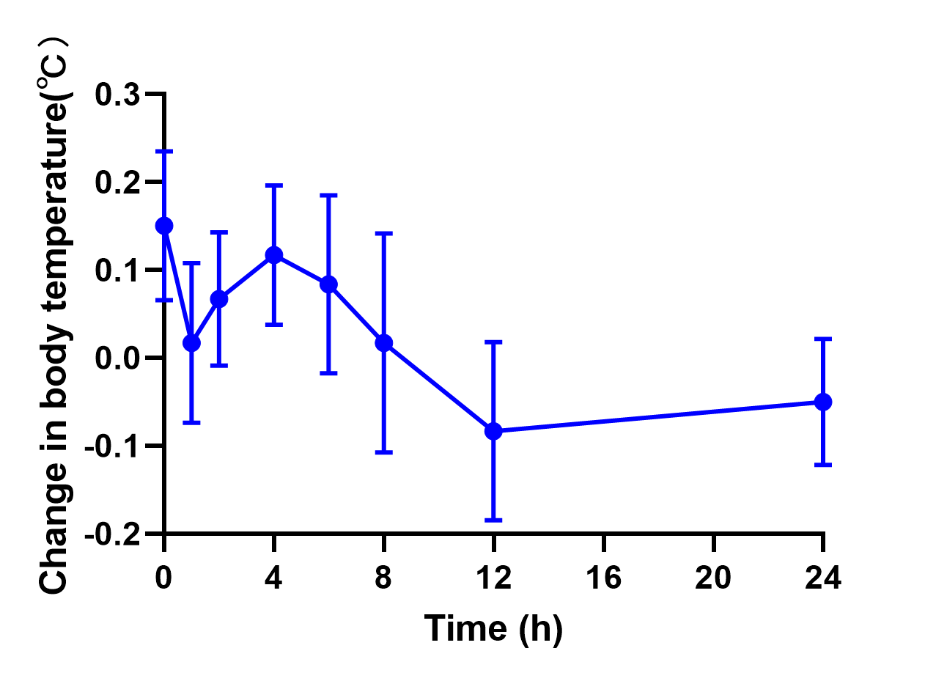


**Supplementary Figure 6.** Effects of PINO on body temperature in mice. Rectal temperatures were measured at 0, 1, 2, 4, 6, 8, 12 and 24 h after intraperitoneal administration of PINO (20 mg/kg). Data are presented as mean ± SEM (n=6).

## Supplementary Tables

**Table S1** Primers for RT-PCR of DRG of mice

| Gene | Forward primer 5’-3’ | Reverse primer 5’-3’ |
| --- | --- | --- |
| *IL-1β* | GCAACTGTTCCTGAACTCAACT | ATCTTTTGGGGTCCGTCAACT |
| *IL-6* | TAGTCCTTCCTACCCCAATTTCC | TTGGTCCTTAGCCACTCCTTC |
| *IL-18* | GACTCTTGCGTCAACTTCAAGG | CAGGCTGTCTTTTGTCAACGA |
| *COX-2* | TTCAACACACTCTATCACTGGC | AGAAGCGTTTGCGGTACTCAT |
| *IFN-γ* | ATGAACGCTACACACTGCATC | CCATCCTTTTGCCAGTTCCTC |
| *TNF-α* | CCTGTAGCCCACGTCGTAG | GGGAGTAGACAAGGTACAACCC |
| *PKA* | AGATCGTCCTGACCTTTGAGT | GGCAAAACCGAAGTCTGTCAC |
| *PKC* | CAGGGTATCTGGGGAATGGC | AGTCCATAATGAGAGGCAGGG |
| *P2X3* | AAAGCTGGACCATTGGGATCA | CGTGTCCCGCACTTGGTAG |
| *CAMK2A* | TGGAGACTTTGAGTCCTACACG | CCGGGACCACAGGTTTTCA |
| *CAMK2B* | CGTTTCACCGACGAGTACCAG | GCGTACAATGTTGGAATGCTTC |
| *18S rRNA* | AGGAATTGACGGAAGGGCACCA | GTGCAGCCCCGGACATCTAAG |

**Table S2** Primers for RT-PCR of RAW264.7

| Gene | Forward primer 5’-3’ | Reverse primer 5’-3’ |
| --- | --- | --- |
| *IL-1β* | GCAACTGTTCCTGAACTCAACT | ATCTTTTGGGGTCCGTCAACT |
| *IL-6* | TAGTCCTTCCTACCCCAATTTCC | TTGGTCCTTAGCCACTCCTTC |
| *IL-18* | GACTCTTGCGTCAACTTCAAGG | CAGGCTGTCTTTTGTCAACGA |
| *COX-2* | TTCAACACACTCTATCACTGGC | AGAAGCGTTTGCGGTACTCAT |
| *TGF-β* | CTCCCGTGGCTTCTAGTGC | GCCTTAGTTTGGACAGGATCTG |
| *TNF-α* | CCTGTAGCCCACGTCGTAG | GGGAGTAGACAAGGTACAACCC |
| *18S rRNA* | AGGAATTGACGGAAGGGCACCA | GTGCAGCCCCGGACATCTAAG |

**Supplementary Video 1**. MD trajectory of PINO-TRPV1 binding. The HHDP group (blue) remains fixed in the pocket, while pinocembrin (green) and glucoside (green) exhibit large fluctuations.
